# Fluorescence-Based Online Monitoring Enables Differentiation in Co-Cultures of Untagged *Streptomyces Species* and *Trichoderma reesei*

**DOI:** 10.1007/s12010-025-05378-y

**Published:** 2025-09-09

**Authors:** Gesa Brauneck, Mareke Sophie Heykena, Dorothea M. Schütterle, Marcel Mann, Ivan Schlembach, Miriam A. Rosenbaum, Jorgen Barsett Magnus

**Affiliations:** 1 AVT – Biochemical Engineering, RWTH Aachen University, Forckenbeckstraße 51, Aachen, 52074 Germany; 2 Leibniz Institute for Natural Product Research and Infection Biology, Hans-Knöll-Institute, Jena, Germany; 3 Faculty of Biological Sciences, Friedrich-Schiller-University Jena, Jena, Germany

**Keywords:** 2D fluorescence spectroscopy, Autofluorescence, Filamentous co-culture, Online monitoring, *Streptomyces*, *Trichoderma reesei*

## Abstract

Microbial co-cultures provide significant advantages over commonly used axenic cultures in biotechnological processes, including increased productivity and access to novel natural products. However, differentiated quantification of the microorganisms in co-cultures remains challenging using conventional measurement techniques. To address this, a fluorescence-based approach was developed to enable the differentiated online monitoring of microbial growth in co-cultures. Co-cultures of *Streptomyces* species (*Streptomyces* sp./spp.) with *Trichoderma reesei* (*T. reesei*) were investigated. *T. reesei* was tagged with the fluorescent protein mCherry, while *Streptomyces* spp. were used as untagged wild-type strains. First, two-dimensional (2D) fluorescence spectra of the individual microorganisms were recorded using an in-house-built device to identify characteristic fluorescence areas for online monitoring of *Streptomyces* spp. These spectra revealed areas of strong autofluorescence for *Streptomyces* spp., while *T. reesei* exhibited little to no autofluorescence, making these areas promising for differentiating the microorganisms in co-cultures. Time-resolved measurements further refined these results, identifying the wavelength combination 405/580 nm as particularly useful for online monitoring the growth of *Streptomyces* spp. To online monitor the co-culture, autofluorescence for *Streptomyces* spp. and mCherry fluorescence for *T. reesei* were used. This approach allowed for differentiated online monitoring of the co-cultures, confirmed by microscopic images. Additionally, the developed method was applied to screen varying inoculation ratios of the microorganisms in co-culture, revealing dynamic changes in the co-culture composition. This highly promising method enables reliable, differentiated online monitoring of co-cultures and has the capacity for expansion to other species, making further validation essential to confirm its potential.

**Supplementary Information:**

The online version contains supplementary material available at 10.1007/s12010-025-05378-y.

## Introduction

In biotechnological research and industrial applications, axenic cultures, consisting of only one microorganism, are still predominantly used. Although this method often provides adequate results for industrial processes, it does not consider natural conditions. In the natural environment, microorganisms occur mostly in complex communities of multiple organisms, showing mutually beneficial or antagonistic interactions. To mimic these natural conditions, co-cultures consisting of two or more microorganisms have increasingly gained attention in biotechnological research [1, 2].

Co-cultures offer several advantages over conventional bioprocesses, enhancing the efficiency and versatility of biotechnological applications, as extensively discussed in numerous review articles [1–3]. This can lead to greater profitability and cost efficiency in biotechnological processes. These advantages primarily include increased productivity of specific products by preventing the formation of inhibitory side products or activating modular metabolic pathways [4–6], the ability to use complex substrates such as lignocellulose [2, 7, 8], and the stimulation of the production of previously undiscovered or hard-to-access compounds, including secondary metabolites [1, 9–12].

Since co-cultures offer high potential for promoting secondary metabolite production, using microorganisms known for their broad secondary metabolite production is particularly promising. One of the best-known genera in this context is *Streptomyces* [13]. Their significance in production for antibiotics, exemplified by streptomycin [14] and neomycin [15], is particularly profound. These soil bacteria exhibit filamentous growth, displaying either a dispersed or pelleted morphology depending on the species and cultivation conditions [16]. *Streptomyces* comprise more than 700 species [17] and are characterized by their enormous genetic diversity as well as a large number of silent gene clusters that can be activated by suitable culture conditions or environmental triggers [13]. Numerous studies demonstrate the potential of co-cultures to activate silent gene clusters and further enhance secondary metabolite production in *Streptomyces* spp. For example, co-cultures of *Streptomyces rochei* and *Rhinocladiella similis* 35 triggered the production of several antimicrobial borrelidins [18]. Co-cultures of *Streptomyces peucetius* and *Aspergillus fumigatus* led to the production of several new natural products, one of which showed activity against human tumor cell lines [9].

However, in addition to the many advantages, co-cultures also have some challenges due to their greater complexity, which could be the reason for their low prevalence in research and industrial applications [1, 2]. Some of these challenges are currently the subject of research. For instance, several studies focus on selecting media and applying process parameters that enable and even control the growth of all microorganisms in a co-culture [19]. Research is also exploring ways to ensure adequate nutrient supply for all microorganisms of the co-culture, prevent the overgrowth of individual organisms, and establish stable co-cultures [19, 20].

Another challenge of co-cultures is the differentiated quantification of the individual microorganisms. Conventional offline and online methods to detect and quantify growth, such as cell dry weight and scattered light, provide a mixed signal that does not differentiate between different microorganisms but rather reflects the growth of all cultivated microorganisms. While some offline methods like sequencing, quantitative polymerase chain reaction, image analysis, and particle size distribution can differentiate microorganisms within co-cultures [21, 22], they require sampling and only offer information at certain time points, often with significant time delays. To gain insights into co-culture dynamics, non-invasive online monitoring techniques are particularly valuable, as they offer high-resolution, real-time data without disrupting cultivation due to sampling. However, such methods are still rare in literature. One promising but operationally complex approach involves recorded microscopy of individual cells in microfluidic chips [23]. As reviewed by Schlembach et al. [24], most existing efforts to online monitor co-cultures rely on spectroscopic techniques, including absorbance [25], scattered light [22], or fluorescence [19, 20, 26]. For instance, Geinitz et al. used scattered light measurement to online monitor a co-culture of *Lactococcus lactis* and *Kluyveromyces marxianus*. However, methods like scattered light and absorbance face the challenge that the signals from both microorganisms are often very similar [22]. A more distinct signal is fluorescence, which can be based on both fluorescent protein tags and autofluorescent molecules, such as vitamins, proteins, and cofactors [19, 20, 26].

This study aims to develop a fluorescence-based online monitoring method to differentiate between microorganisms in co-cultures of untagged *Streptomyces* spp. and *T. reesei*. *Streptomyces* spp. were chosen for their significant potential in secondary metabolite production, while *T. reesei* was selected as the co-culture partner because its interaction with *Streptomyces* spp. closely mimics the natural interactions found in forest soils. Previous studies have already explored co-cultures of *T. reesei* and the model organism *Streptomyces coelicolor* (*S. coelicolor*) on cellulose, revealing population dynamics associated with increased secondary metabolite production and strategies to tune these dynamics for enhanced production. In these studies, both microorganisms were tagged using fluorescent proteins, which limited the application to other *Streptomyces* spp. [19, 20]. The current objective is to expand the method’s applicability for screening by using different *Streptomyces* spp. Thereby, *T. reesei* remains tagged with the fluorescent protein mCherry, whereas untagged wild-type *Streptomyces* spp. are now used.

## Materials and Methods

### Microorganisms

The following *Streptomyces* spp. were received from the Hans-Knöll-Institute culture collection (Jena, Germany): *Streptomyces albofaciens* DSMZ 40268, *Streptomyces alboniger* DSMZ 40043, *Streptomyces bobili* DSMZ 40481 (*S. bobili*), formerly identified as *Streptomyces galilaeus* [27], *Streptomyces coeruleorubidus* DSMZ 40145 (*S. coeruleorubidus*), *Streptomyces fradiae* DSMZ 40063, *Streptomyces rimosus* DSMZ 40260, and *Streptomyces venezuelae* DSMZ 40230. For *Streptomyces* spp., spore solutions were prepared similarly to Hobbs et al. [16] and Finger et al. [28]. A spore solution was spread on agar plates, consisting of 20 g L^−1^ soy flour (Reformhaus, Zarrentin, Germany), 20 g L^−1^ mannitol, and 20 g L^−1^ agar (both from Carl Roth GmbH + Co. KG, Karlsruhe, Germany). After incubation at 30 °C for 7 to 10 days, depending on the species, spores were harvested by scraping off and suspending them in deionized water.

The construction of the tagged strain *T. reesei* RUT-C30 mCherry was previously reported [20]. The spores of *T. reesei* were produced by cultivating them in modified Pakula medium, see section “Medium Composition,” with an altered carbon source composition of 5 g L^−1^ glucose and 30 g L^−1^ α-cellulose (Sigma-Aldrich, St. Louis, USA).

For all spore solutions, mycelium was removed by filtration with a 40-µm cut-off cell strainer (pluriSelect Life Science, Leipzig, Germany). The spore concentration was measured using a particle sizer (Multisizer 4e, Beckmann Coulter, Brea, United States) and adjusted to 10^8^ mL^−1^ spores. The spores were stored at − 80 °C, in 25% glycerol (Carl Roth GmbH + Co. KG, Karlsruhe, Germany) for *Streptomyces* spp. and in deionized water for *T. reesei*.

### Medium Composition

All cultivations were performed in a modified Pakula medium [29], which was adapted similarly to the media used by Antonov et al. [30] and Finger et al. [28]. All media components were sourced from either Carl Roth GmbH + Co. KG (Karlsruhe, Germany), Merck KGaA (Darmstadt, Germany), or VWR International (Radnor, USA). If not stated otherwise, the medium consists of 15 g L^−1^ glucose, 0.1 M 2-(N-morpholino)-ethanesulfonic acid, 7.6 g L^−1^ (NH_4_)_2_SO_4_, 2.6 g L^−1^ KH_2_PO_4_, 0.50 g L^−1^ MgSO_4_ × 7H_2_O, 0.45 g L^−1^ citric acid, 0.30 g L^−1^ urea, 0.23 g L^−1^ CaCl_2_ × 2H_2_O, 0.05 g L^−1^ NaCl, 0.04 g L^−1^ ZnSO_4_ × 7H_2_O, 8.0 mg L^−1^ CuSO_4_ × 5H_2_O, 6.8 mg L^−1^ CoCl_2_ × 6H_2_O, 6.0 mg L^−1^ Fe_2_(SO_4_)_3_xH_2_O, 4.0 mg L^−1^ MnSO_4_ × 7H_2_O, 2.0 mg L^−1^ H_3_BO_3_, and 0.01% v/v Tween-80. The pH value of the medium was adjusted to 6.7 using 5 M NaOH (Carl Roth GmbH + Co. KG, Karlsruhe, Germany), and Tween-80 was added after pH value adjustment. The medium was then sterile filtered using a 0.22-µm cut-off filter (Millipore, Merck KGaA, Darmstadt, Germany). The medium was stored as a twofold concentrated stock solution at room temperature, with glucose and 2-(N-morpholino)-ethanesulfonic acid added freshly before each experiment.

### Precultures

For precultures, modified Pakula medium, see section “Medium Composition,” was inoculated with 10^6^ mL^−1^ spores. Precultures were performed in unbaffled 250-mL shake flasks with a filling volume of 20 mL, at a temperature of 30 °C, a humidity of 80%, a shaking frequency of 350 rpm, and a shaking diameter of 50 mm (Climo-Shaker ISF1-X, Adolf Kühner AG, Birsfelden, Switzerland). To prevent pellet formation and reach a higher homogeneity in the precultures of *Streptomyces* spp., 600 mg of glass beads with a diameter of 2.7 ± 0.27 mm (BioSpec Products, Bartlesville, USA) was added to each flask [31]. All precultures were cultivated for 3 days. After cultivation, the cells were harvested by centrifugation at 4000 rpm for 10 min (Rotina 35R, Andreas Hettich GmbH, Tuttlingen, Germany), the supernatant was discarded, and the cell pellet was resuspended in deionized water.

### Main Cultures and Online Monitoring Devices

Main cultures were performed in modified Pakula medium, see section “Medium Composition,” and inoculated with a defined optical density at the beginning of the cultivation (OD_start_), measured at 600 nm in a photometer (Genesys 20, Thermo Fisher Scientific, Darmstadt, Germany). For axenic cultures, the OD_start_ was set to 0.2. For co-cultures, the OD_start_ was varied for both microorganisms to set different inoculation ratios, see Table 1.

**Table 1:** Inoculation ratios of co-cultures of *Streptomyces* spp. and *T. reesei* for main cultures. The inoculation ratio was set by varying the OD_start_ of both microorganisms from 0 to 0.2. The OD_start_ of one microorganism was kept constant with the reference, the axenic culture

Inoculation ratio of co-culture *Streptomyces* spp.:*T. reesei*	OD_start_ of *Streptomyces* spp.	OD_start_ of *T. reesei*
1:0 (axenic)	0.2	0
1:0.1	0.2	0.02
1:0.2	0.2	0.04
1:0.5	0.2	0.1
1:1	0.2	0.2
0.5:1	0.1	0.2
0.2:1	0.04	0.2
0.1:1	0.02	0.2
0:1 (axenic)	0	0.2

All main cultures, regardless of the used device, were performed in 48-well round-well microtiter plates (MTP-R48-B, Beckmann Coulter, Brea, USA), sealed with gas permeable foils (HJ-BIOANALYTIK GmbH, Erkelenz, Germany). The main cultures were conducted with a filling volume of 1 mL, at a temperature of 30 °C, a shaking frequency of 800 rpm, and a shaking diameter of 3 mm (Climo-Shaker ISF1-X, Adolf Kühner AG, Birsfelden, Switzerland).

For recording 2D fluorescence spectra, an in-house-built device that measures fluorescence intensity in arbitrary units (A. U.) of all excitation and emission wavelength combinations in a defined range was used [32]. Excitation was measured from 280 to 700 nm with a step size of 5 nm, and emission between 270 and 700 nm with a step size of 1 nm. The integration time was set to 900 ms. The area within 30 nm around the scattered light (same excitation and emission wavelength) was cut off. Data was referenced to the first measuring cycle at the beginning of the cultivation.

For all other main cultures, another in-house-built device that combines a BioLector system with measurement of the respiration activity (µRAMOS-BioLector-combination) was used [33, 34]. The oxygen transfer rate (OTR) and fluorescence intensity of specific wavelength combinations that are specified in 3 were measured. For fluorescence measurements, the integration time was set to 900 ms.

### Offline Measurements

Microscopic images of the culture broth were taken with a light microscope (Nikon Eclipse E600, Nikon Instruments, Tokyo, Japan), equipped with a camera (Sony α 6000, Sony, Tokyo, Japan) and a 10 × objective, providing a total magnification of × 100. For sample preparation, 15 µL of culture broth was pipetted onto a microscope slide and covered with a coverslip. Images were taken at the end of the cultivation.

To determine the cell dry weight gravimetrically, the whole culture broth of one well was centrifuged for 10 min at 14,000 rpm (Sigma 1-15PK, Sigma Laborzentrifugen GmbH, Osterode, Germany) in a pre-weighed reaction vessel, and the supernatant was removed. The reaction vessel containing the cell pellet was then dried at 80 °C for 48 h and weighed again to determine the cell dry weight from the weight difference.

The glucose concentration was quantified using high-performance liquid chromatography (HPLC). The supernatant after centrifugation for cell dry weight determination was subsequently sterile filtered using a 0.2-µm cut-off filter (VWR International, Radnor, USA) and stored at − 20 °C until the measurement. HPLC analysis was performed with a Thermo Fisher Ultimate 3000 (Thermo Fisher Scientific Inc., Waltham, USA), equipped with an ERC RefractoMax 520 detector (IDEX Health & Science LLC, Oak Harbor, USA) and a Rezex ROA‐Organic Acid column (300 × 7.8 mm, Phenomenex, Torrance, USA). The flow rate of the mobile phase, 25 mM H_2_SO_4_ (Carl Roth GmbH + Co. KG, Karlsruhe, Germany), was set to 0.8 mL min^−1^ at a column temperature of 85 °C.

## Results and Discussion

### Identifying Suitable Fluorescence Wavelength Combinations for Online Monitoring of *Streptomyces* spp.

**Fig. 1:**
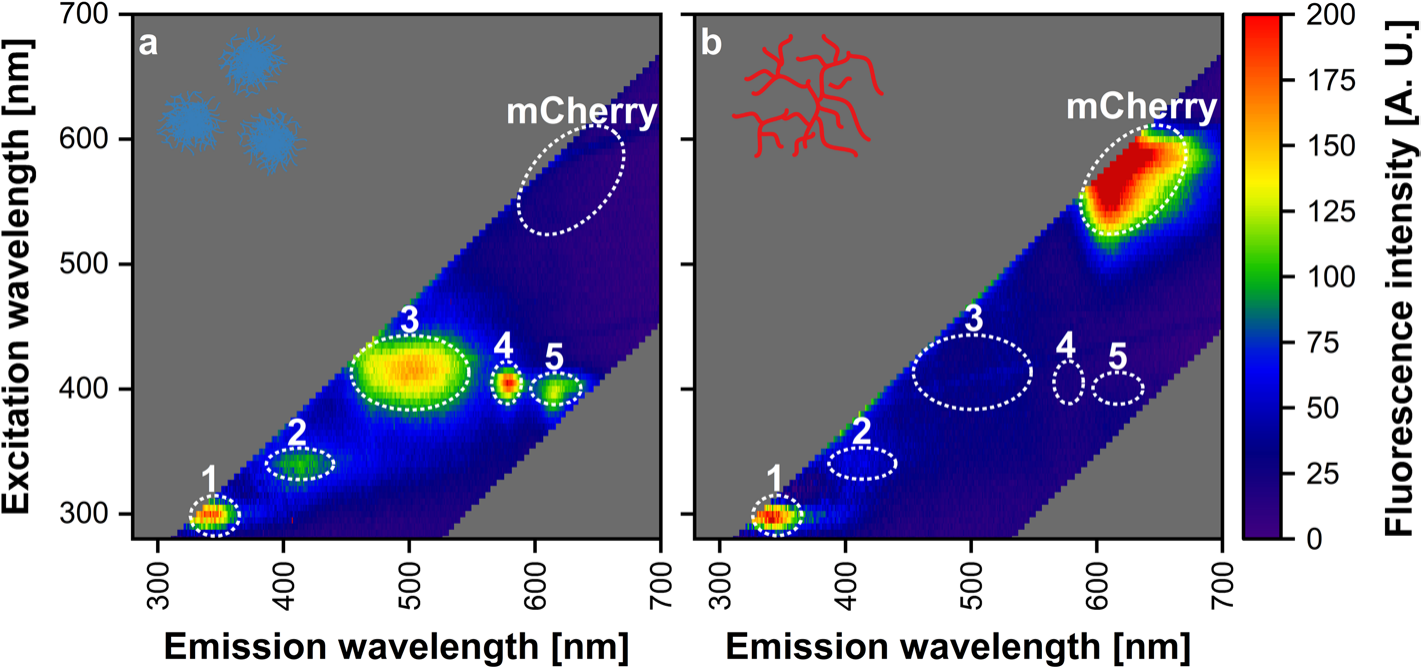
Differential 2D fluorescence spectra of axenic cultures of *S. coeruleorubidus* (**a**) and *T. reesei* RUT-C30 mCherry (**b**). Display of fluorescence intensity depending on different excitation and emission wavelength combinations as a contour plot. The spectra were recorded at the end of the growth phase shortly before glucose of the respective cultivation was depleted. Cultivation was conducted in a 48-well round-well microtiter plate within the in-house-built device that measures fluorescence intensity of all excitation and emission wavelength combinations in a defined range [32]. Spectroscopic measurement settings: excitation wavelength range = 270–700 nm, excitation step size = 5 nm, emission wavelength range = 270–700 nm, emission step size = 1 nm, integration time = 900 ms. The area 30 nm around the scattered light (same excitation and emission wavelength) was cut off. Data was referenced to the first measuring cycle at the beginning of the cultivation. Unmeasured areas are shown in gray. Selected Areas 1 to 5 and mCherry are marked with white, dotted circles

The first step was to select suitable wavelength combinations that allow for differentiated online monitoring of the growth of different microorganisms in co-culture using fluorescence. *T. reesei* was tagged with the fluorescence protein tag mCherry, which enabled easy growth detection. For *Streptomyces* spp., wild-type strains without a fluorescent protein tag were used to enable screening of a broad range of *Streptomyces* spp., eliminating the need for the additional effort of tagging with fluorescent proteins. This facilitates immediate application, for example, co-culture screening. Therefore, autofluorescent molecules should be used to online monitor these species. To find suitable wavelength combinations, 2D fluorescence spectra of axenic cultures of both microorganisms were recorded in an in-house-built device [32], see differential spectra in Fig. 1. The 2D fluorescence spectra were recorded at the end of the exponential growth phase to identify areas that exhibit a strong correlation with microbial growth.

The differential 2D fluorescence spectrum of one *Streptomyces* sp., *S. coeruleorubidus*, is shown in Fig. 1a. The spectrum reveals five areas with high fluorescence intensity which are marked as Area 1 (excitation 280 to 315 nm, emission 325 to 365 nm), Area 2 (excitation 330 to 415 nm, emission 385 to 440 nm), Area 3 (excitation 385 to 445 nm, emission 455 to 550 nm), Area 4 (excitation 390 to 425 nm, emission 565 to 590 nm), and Area 5 (excitation 385 to 415 nm, emission 600 to 640 nm). Figure 1b shows the differential 2D fluorescence spectrum of the mCherry-tagged *T. reesei* with the characteristic strong mCherry fluorescence at an excitation of 530 to 630 nm and at an emission of 590 to 690 nm. The wavelength combination usually measured for this fluorescent protein is 587/610 nm [35], which is exactly in the area observed here. Compared to the spectrum of *S. coeruleorubidus*, *T. reesei* also shows high fluorescence in Area 1, making this area unsuitable for differentiation between the species. Comparison with the literature shows that 2D fluorescence spectra of several microorganisms have already been recorded, with fluorescence caused by the aromatic amino acids of proteins typically observed in this area [36, 37]. The fluorescence intensity in Areas 2 to 5 is very low or not detectable for *T. reesei*, suggesting that these areas are likely specific to *S. coeruleorubidus* or possibly to *Streptomyces* spp. in general. To confirm this, 2D fluorescence spectra of other *Streptomyces* spp. were recorded, see Supplement 1. The differential 2D fluorescence spectra of all investigated *Streptomyces* spp. closely resemble that of *S. coeruleorubidus*, as the fluorescence intensity maxima observed in Areas 1 to 5 are also present here. Depending on the *Streptomyces* sp., only the intensity of these fluorescence areas varies. Palacio-Barrera et al. also recorded 2D fluorescence spectra of *S. coelicolor*, identifying a fluorescence intensity maximum comparable to the observed Area 3 [20].

The identified Areas 2 to 5 are further compared with the literature to determine whether the observed fluorescence could be due to known autofluorescent molecules. First, the comparison is made with molecules that are well-known for their autofluorescent properties and are commonly measured, such as nicotinamide adenine dinucleotide (NADH) and flavin adenine dinucleotide (FAD). Only a slight fluorescence intensity is observed for these molecules in cultures of both *T. reesei* and *Streptomyces* spp., and the differential 2D fluorescence spectra show no overlap with the observed Areas 2 to 5 [36, 38, 39]. For pyridoxine, another common autofluorescent molecule, the typical excitation and emission wavelengths of 330 nm and 390 nm, respectively, overlap with the observed Area 2, which suggests that the observed high fluorescence intensity is due to pyridoxine [39, 40]. Ödman et al. even observed high pyridoxine fluorescence specifically for *S. coelicolor* [41]. In their publication, Cox and Adams reported the fluorescence of pyoverdine, a siderophore, at 400/460 nm, which they used for its quantification [42]. Similarly, Palanché et al. observed high fluorescence intensities for an anthryl-desferriferrichrome analogue, also a siderophore, at emission wavelengths ranging from 450 to 500 nm following excitation at 416 nm [43]. In both studies, the fluorescence wavelengths of the siderophores closely resemble those observed in Area 3, suggesting that this fluorescence could be related to the presence of siderophores. Furthermore, several publications showed that porphyrins, including protoporphyrin IX and hematoporphyrin, exhibit fluorescence with an excitation near 400 nm and emission at around 580 and 600 nm [40, 44–46]. This closely aligns with the fluorescence of the observed Areas 4 and 5, indicating that porphyrins might be responsible for the detected fluorescence areas. For definitive identification, additional analysis of the fluorescent molecules, which is beyond the scope of this work, would be required.

### Time-Resolved Comparison of Selected Wavelength Combinations

In section “Identifying Suitable Fluorescence Wavelength Combinations for Online Monitoring of *Streptomyces* spp.,” distinct areas were identified that were characteristic for one of the two microorganisms of the co-culture: the mCherry area for *T. reesei* and Areas 2 to 5 for *Streptomyces* spp. The next step involved examining the correlation between fluorescence intensity and growth to determine the most suitable wavelength combinations for microorganism-specific online monitoring. To achieve this, axenic cultures of both microorganisms were cultivated in the µRAMOS-BioLector-combination, where the OTR was measured alongside specifically defined wavelength combinations corresponding to the observed fluorescence areas. The total oxygen consumption, represented by the integral of the OTR, was used as a measure of biomass. The process investigated in this study involves only biomass formation without any product formation, meaning that all consumed substrate, including oxygen, is used for biomass growth. A correlation between total oxygen consumption and biomass has been previously reported in the literature for various cell types, including microbial cells [47, 48]. This approach offers advantages over commonly used growth detection techniques like cell dry weight, as it facilitates online monitoring and helps mitigate errors that arise from unreliable and non-reproducible results. These errors are particularly associated with commonly used methods, especially those influenced by the morphological effects of cultures with filamentous microorganisms, as used in this study. Since all *Streptomyces* spp. exhibited comparable differential 2D fluorescence spectra, subsequent experiments focused on two representative species: *S. coeruleorubidus* and *S. bobili*.

Initially, it was confirmed that total oxygen consumption could be used to monitor biomass and estimate the timing of the exponential growth phase by comparing different online and offline data of an axenic *S. coeruleorubidus* cultivation, see Supplement 2. Following a brief lag phase, the total oxygen consumption increases exponentially up to 24 h, indicating the phase of exponential growth. The OTR is also shown, which exhibits a sharp drop from 26 to 7 mmol L^−1^ h^−1^ after 24 h. At that time, glucose is completely consumed according to HPLC measurements, which leads to the end of exponential growth. The low OTR of 3 mmol L^−1^ h^−1^ observed afterward leads to a slight increase in total oxygen consumption, which can be explained by potential secondary processes, such as sporulation, which has been reported as oxygen-dependent for different filamentous, spore-forming microorganisms [49, 50]. It can thus be concluded that total oxygen consumption provides a reliable indication of when glucose is consumed, marking the end of the exponential biomass growth phase. In contrast, the cell dry weight exhibits an irregular trend, marked by a decrease during the exponential growth phase and substantial standard deviations. As previously noted, the cell dry weight results indicate a high susceptibility to error, which is further corroborated by the data. Consequently, total oxygen consumption will be used as a more reliable measure of biomass growth in subsequent analyses.

**Fig. 2:**
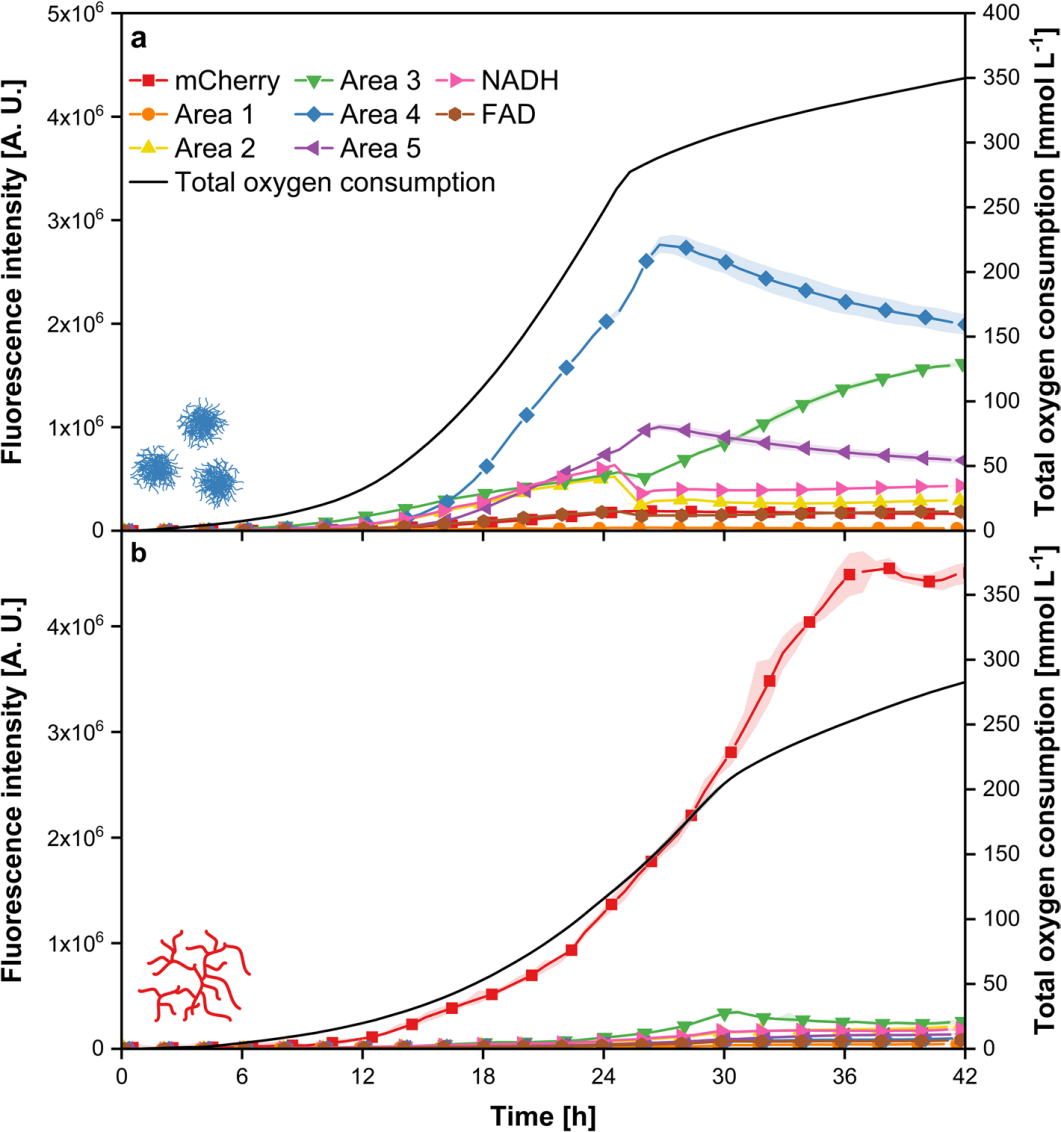
Time-resolved fluorescence measurement of selected fluorescence wavelength combinations of axenic cultures of *S. coeruleorubidus* (**a**) and *T. reesei* RUT-C30 mCherry (**b**). Cultivation was conducted in a 48-well round-well microtiter plate within the µRAMOS-BioLector-combination [33, 34]. Spectroscopic measurement settings: integration time = 900 ms, Area 1: 280/350 nm, Area 2: 340/420 nm, Area 3: 400/470 nm, Area 4: 405/580 nm, Area 5: 400/630 nm, NADH: 340/460 nm, FAD: 340/530 nm, mCherry: 587/610 nm. For clarity, only every third data point of fluorescence intensity is plotted as a symbol. Lines are drawn through all data points. The data presented are mean values derived from biological triplicates for each measurement. The shaded areas indicate the standard deviations

Figure 2a shows the previously described course of the total oxygen consumption and the time-resolved course of fluorescence intensities of *S. coeruleorubidus*. When examining fluorescence intensity, a noticeable difference in signals emerges for the various wavelength combinations. The highest fluorescence intensity is recorded for Area 4 (405/580 nm), where intensity increases until 24 h, with a distinct bend in the curve at that time. Afterward, it rises again for approximately 2 h up to 2.8 × 10^6^ A. U. before slightly decreasing toward the end of the cultivation. The fluorescence intensity in Area 5 (400/630 nm) follows a similar course but remains lower throughout the entire cultivation. Fluorescence intensities of all other observed fluorescence areas also increase during the first 24 h, although these values are considerably lower than in Area 4 with values well below 1 × 10^6^ A. U. Thus, the fluorescence intensity of some wavelength combinations is in a range that no longer notably differs from the fluorescence intensity of *T. reesei*, making these areas unsuitable for differentiated online monitoring in co-cultures. The change in fluorescence intensity after 24 h coincides with the time point of glucose consumption and the end of exponential growth in total oxygen consumption, suggesting a correlation between these two measurables. To assess this correlation, fluorescence intensity was plotted against total oxygen consumption during the exponential growth phase, see Supplement 3a. Due to the high intensity and similar trends between fluorescence and total oxygen consumption, fluorescence in Area 4 is considered the most promising for online monitoring of *S. coeruleorubidus*, so it is used for the plot. This plot reveals a linear correlation between fluorescence intensity and total oxygen consumption during exponential growth, indicating that fluorescence in Area 4 is suitable for online monitoring of the biomass growth of *S. coeruleorubidus*. To test the applicability to other *Streptomyces* spp., similar cultivations were conducted using *S. bobili*, see Supplement 4. The overall fluorescence intensity for *S. bobili* is lower than that for *S. coeruleorubidus,* but depending on the wavelength combination, it is notably higher than the fluorescence intensity of *T. reesei*. While the fluorescence intensity in Area 3 for *S. coeruleorubidus* showed a course very similar to total oxygen consumption, the one observed for *S. bobili* was notably different. The fluorescence intensity increases sharply during the lag phase, where a delay in OTR is also visible, see Supplement 5. It then reaches a plateau during the growth phase, followed by an increase again, indicating that this area is unsuitable for online monitoring of this species. Similar to *S. coeruleorubidus*, the highest fluorescence intensity for *S. bobili* was observed in Area 4 with up to 0.6 × 10^6^ A. U. during the exponential growth phase, and a linear correlation with total oxygen consumption was also confirmed, see Supplement 6. This suggests that online monitoring in co-culture using the fluorescence intensity of Area 4 should also be feasible for this *Streptomyces* sp.

As depicted in Fig. 2b, for the mCherry-tagged *T. reesei*, the total oxygen consumption increases exponentially after a short lag phase during the first 29 h of cultivation. After that, a slight increase in total oxygen consumption is observed. The possible reason for this, sporulation, has been discussed before for *S. coeruleorubidus*. When looking at the fluorescence intensities, the mCherry signal (587/610 nm) in particular stands out due to its high intensity. After a short lag phase, the fluorescence intensity of mCherry increases exponentially during the first 29 h up to 2.6 × 10^6^ A. U., after which a turning point can be observed in the curve, which is accompanied by an increasing standard deviation. The signal increases until 36 h up to 4.5 × 10^6^ A. U. While sporulation may also affect the mCherry signal, the continued increase after the glucose consumption and end of growth could also result from cell lysis, leading to the release of mCherry, as already described by Schlembach and Ott [51]. Nevertheless, due to the very similar trends observed during the exponential growth phase, a plot of fluorescence intensity versus total oxygen consumption was created, revealing a linear correlation, see Supplement 3b. It is also worth noting that very low mCherry fluorescence intensities were recorded for both *Streptomyces* spp., eliminating the possibility of overlapping with this signal and making it suitable for *T. reesei* online monitoring in co-culture. For all other measured wavelength combinations, the fluorescence intensities are very low compared to the mCherry fluorescence and the intensities observed for *Streptomyces* spp. with values well below 1 × 10^6^ A. U., as already seen in the differential 2D fluorescence spectra. This makes these wavelength combinations, particularly the previously described Area 4, promising for online monitoring of *Streptomyces* spp.

### Online Monitoring of Co-Cultures with Selected Wavelength Combinations

For online monitoring of the co-cultures, two fluorescence wavelength combinations were used, which were identified as the most suitable in sections “Identifying Suitable Fluorescence Wavelength Combinations for Online Monitoring of *Streptomyces* spp.” and “Time-Resolved Comparison of Selected Wavelength Combinations”: mCherry (587/610 nm) for *T. reesei* and Area 4 (405/580 nm) for *Streptomyces* spp. Finger et al. have previously demonstrated that the growth of co-cultures can be controlled through various parameters, including the inoculation ratio of the two microorganisms of the co-culture [19]. Based on this, co-cultures were set up with different inoculation ratios. Therefore, one of the two microorganisms was adjusted to have the same OD_start_ as the corresponding reference, the axenic culture. This ensured that the biomass for this microorganism was similar to the reference culture in the beginning, eliminating any potential effects due to differences in initial biomass. The OD_start_ of the other microorganism was reduced to 10%, resulting in much lower biomass in the beginning.

**Fig. 3:**
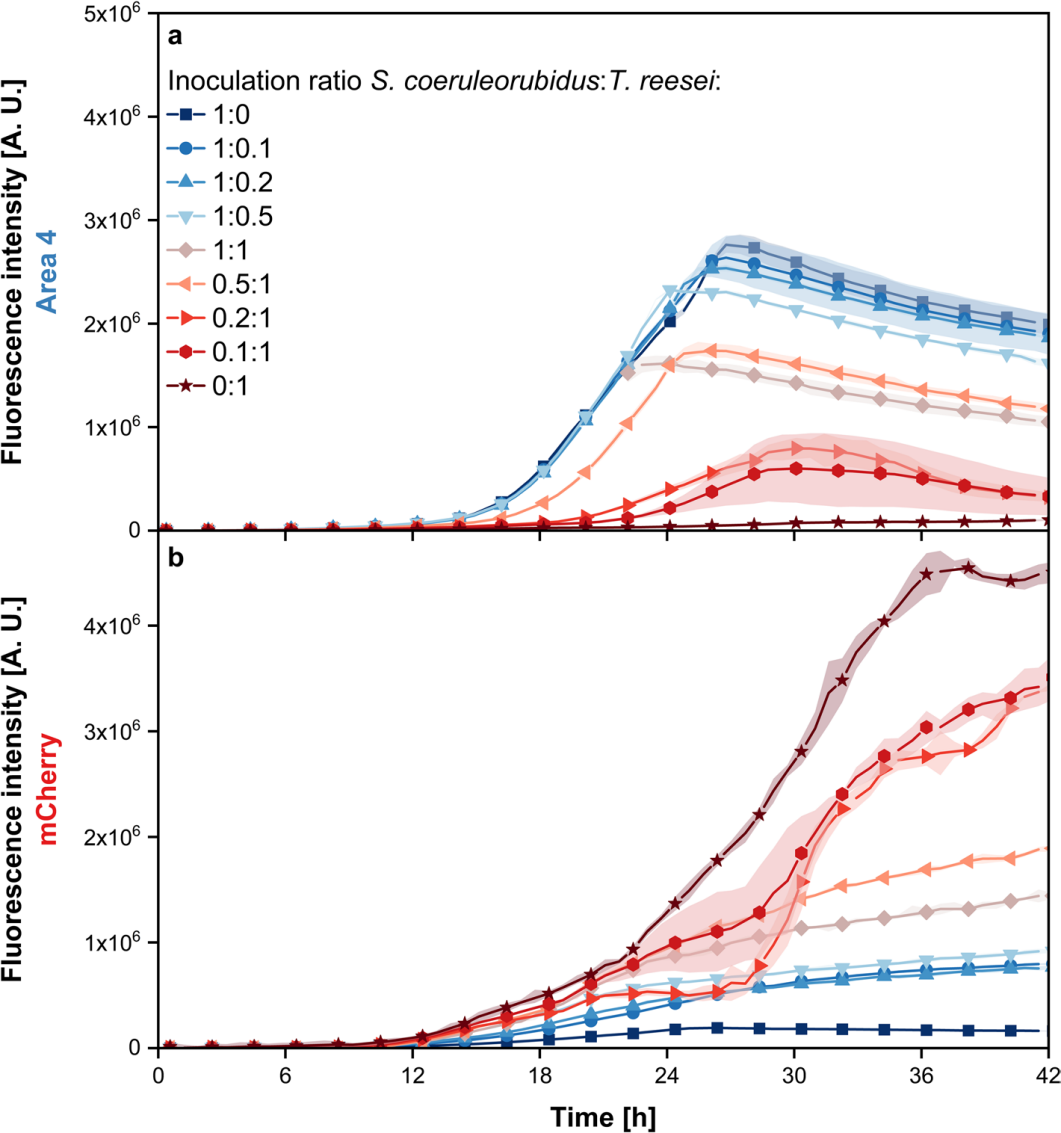
Time-resolved fluorescence measurement of Area 4 (**a**) and mCherry (**b**) of co-cultures of *S. coeruleorubidus* and *T. reesei* RUT-C30 mCherry with varying inoculation ratios. Cultivation was conducted in a 48-well round-well microtiter plate within the µRAMOS-BioLector-combination [33, 34]. Spectroscopic measurement settings: integration time = 900 ms, Area 4: 405/580 nm, mCherry: 587/610 nm. For clarity, only every third data point is plotted as a symbol. Lines are drawn through all data points. The data presented are mean values derived from biological triplicates for each measurement. The shaded areas indicate the standard deviations

Figure 3 shows the fluorescence intensity courses for the different inoculation ratios of *S. coeruleorubidus* and *T. reesei*. The course of the two axenic cultures (1:0 and 0:1), which serve as reference cultures, was already discussed in detail in section “Time-Resolved Comparison of Selected Wavelength Combinations.” For the co-cultures with inoculation ratios favoring *S. coeruleorubidus* (1:0.1, 1:0.2, and 1:0.5), a high fluorescence intensity in Area 4 is observed throughout the whole cultivation. At the end of the exponential growth phase, identified by the shift in the curve as well as OTR, see Supplement 7, the fluorescence intensity ranges from 1.7 to 2.1 × 10^6^ A.U., which is only slightly lower than that of the axenic reference culture. For the same cultures, mCherry fluorescence intensities are measured, which, although higher than the axenic *S. coeruleorubidus* reference culture, are still notably lower than the fluorescence intensity observed in the axenic *T. reesei* reference culture. For the two co-cultures with inoculation ratios of 1:1 and 0.5:1, the fluorescence intensities for both measured wavelength combinations, mCherry and Area 4, are approximately half of those observed in the axenic reference cultures at the end of the cultivation, indicating a balanced co-culture with each microorganism contributing roughly equally. Additionally, it can be observed that the co-cultures with inoculation ratios of 1:0.5 and 1:1 reach the end of the exponential growth phase earlier than the axenic *S. coeruleorubidus* reference culture, specifically after approximately 22 h. This can be explained by the fact that these cultures contain substantially more cells—up to twice as many as the reference cultures—since both microorganisms were inoculated at a high OD_start_. The two co-cultures with a significantly higher *T. reesei* inoculation ratio (0.2:1 and 0.1:1) show lower fluorescence intensities in Area 4 compared to the other co-cultures and the axenic *S. coeruleorubidus* reference culture. However, the mCherry signal is much higher, suggesting that these co-cultures are dominated by *T. reesei*. The applicability to co-cultures with other *Streptomyces* spp. was tested using *S. bobili*, see Supplement 8. In these co-cultures, it is also visible that there is an effect of varying inoculation ratios on the different time-resolved fluorescence intensities, leading to a dominance of the microorganism with higher OD_start_. In contrast to the co-culture with *S. coeruleorubidus*, no strong dominance of *S. bobili* was observed in the co-cultures with lower inoculation ratio gradients (1:0.5, 1:1, and 0.5:1). This can be attributed to the fact that this *Streptomyces* spp. exhibits a longer lag phase, which is visible especially in the axenic culture, see Supplement 5.

Since significant differences in the fluorescence signals were observed when testing the method for online monitoring for varying inoculation ratios, the co-culture ratios will be determined and compared. To achieve this, a comparison of fluorescence intensity over time will be made between the co-cultures and the axenic cultures, using the fluorescence at the end of the exponential growth phase of the axenic culture as a reference (100%). Therefore, the following assumptions are made: In all axenic and co-cultures, the same amount of carbon source, in this case glucose, was added. Since the other nutrients were provided in excess, glucose is the only growth-limiting substrate. It is assumed that nearly all glucose is used for biomass growth, either for the growth of *Streptomyces* spp. or for the growth of *T. reesei*. Therefore, at the end of the cultivation, the total signal from both microorganisms of the co-culture should represent nearly 100%. For the subsequent validation of the results, it was determined that a deviation of 20% from the total signal would be considered a tolerable outcome. The calculations are mathematically described by Eqs. (1) and (2).

1 $${\mathrm{Ratio}}_{Streptomyces\;\mathrm{sp}.}\left[\%\right]=\frac{{\mathrm I}_{\mathrm{Area}\;4\;\mathrm{of}\;\mathrm{co}-\mathrm{culture}}\left[\mathrm A.\;\mathrm U.\right]}{{\mathrm I}_{\mathrm{Area}\;4\;\mathrm{of}\;Streptomyces\;\mathrm{sp}.\;\mathrm{axenic},\;\mathrm{end}}\left[\mathrm A.\;\mathrm U.\right]}$$

2 $${\mathrm{Ratio}}_{T.reesei}\left[\%\right]\frac{{\mathrm I}_{\mathrm{mCherryofco}-\mathrm{culture}}\lbrack\mathrm A.\mathrm U{.\rbrack}}{{\mathrm I}_{\mathrm{mCherry}\operatorname{of}\;\mathrm T.\mathrm{reesei}\;\mathrm{axenic},\mathrm{end}}\lbrack\mathrm A.\mathrm U.\rbrack}$$

**Fig. 4:**
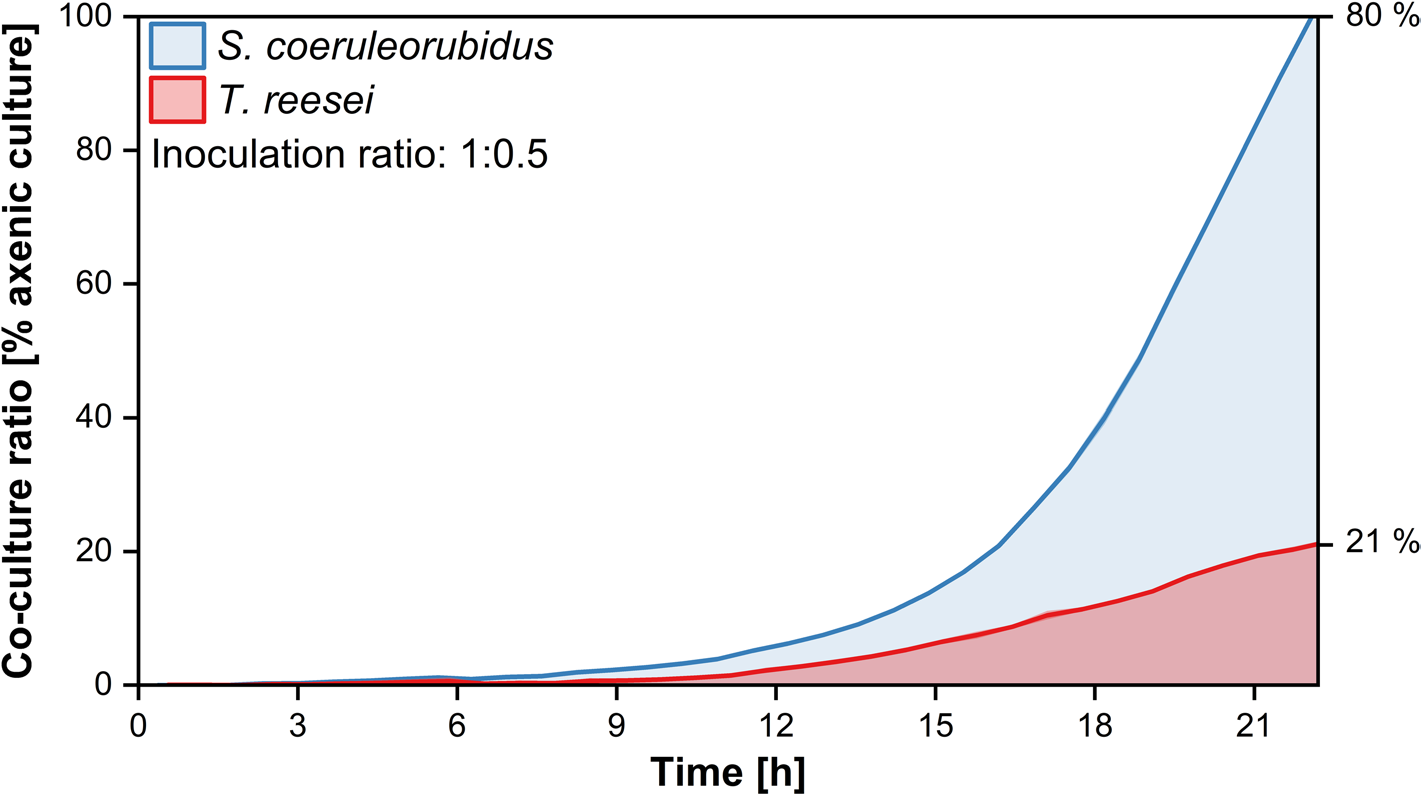
Time-resolved co-culture ratio compared to axenic cultures for co-culture of *S. coeruleorubidus* and *T. reesei* RUT-C30 mCherry with inoculation ratio 1:0.5. Calculation of co-culture ratios according to Eqs. (1) and (2). Cultivation was conducted in a 48-well round-well microtiter plate within the µRAMOS-BioLector-combination [33, 34]. Spectroscopic measurement settings: integration time = 900 ms, Area 4: 405/580 nm, mCherry: 587/610 nm. The data presented are mean values derived from biological triplicates for each measurement. The shaded areas indicate the standard deviations. Only data of the growth phase until glucose depletion is shown

The time-resolved development of the co-culture ratios of *S. coeruleorubidus* and *T. reesei* is exemplified by the co-culture with an inoculation ratio of 1:0.5 in Fig. 4. The development of the co-culture ratio reveals almost no increase of the signal for either microorganism during the first 6 to 9 h. After approximately 9 h, an increase in signal for *S. coeruleorubidus* is observed, followed by a slight delay before the signal for *T. reesei* also rises. Throughout the entire cultivation, *S. coeruleorubidus* dominates, with the co-culture reaching approximately 80% of the fluorescence signal compared to the axenic culture for *S. coeruleorubidus* and 21% for *T. reesei* at the end of the exponential growth phase. The course of all other inoculation ratios is displayed in Supplement 9.

**Fig. 5:**
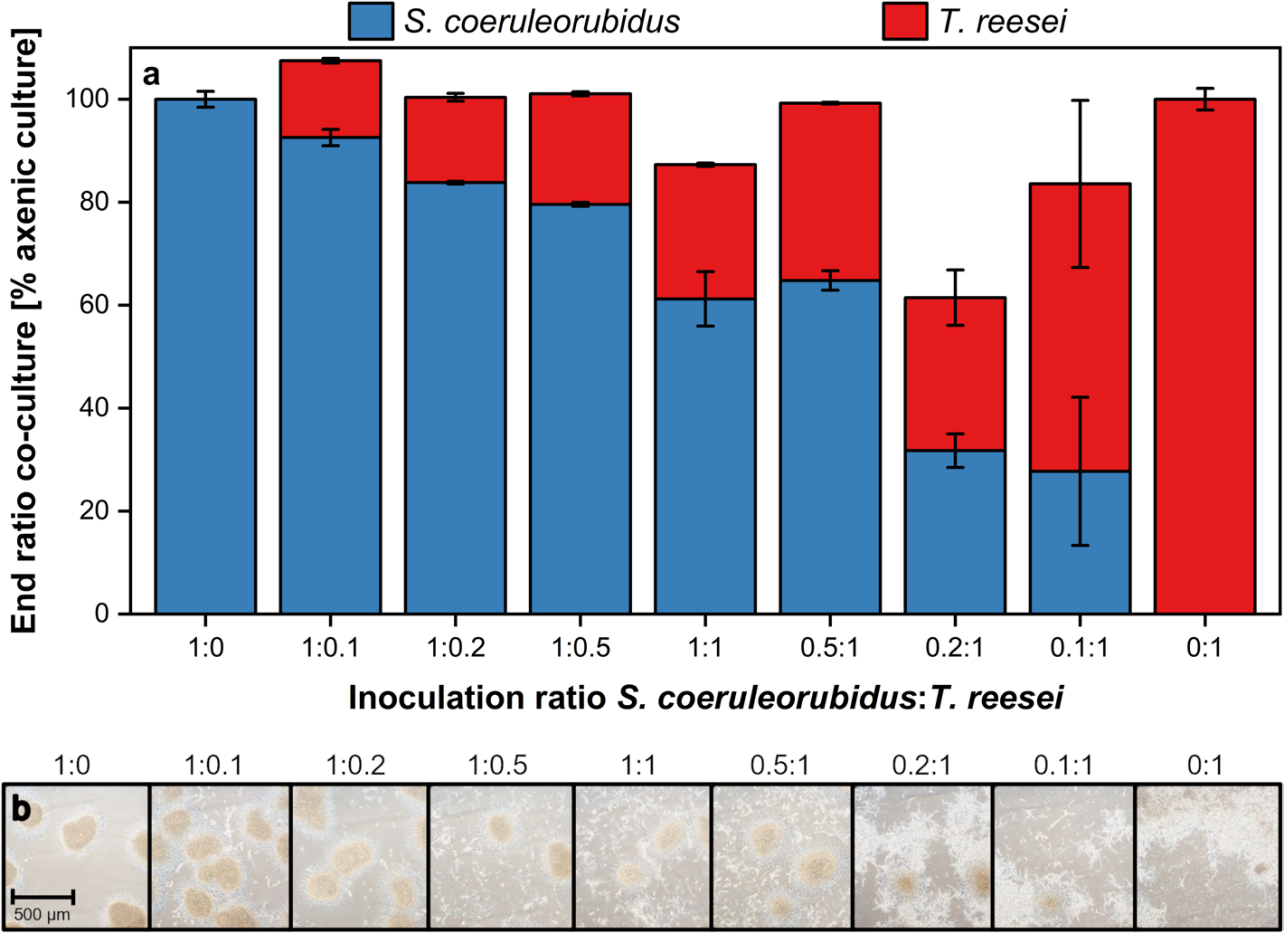
End ratios (**a**) and microscopic images (**b**) of co-cultures of *S. coeruleorubidus* and *T. reesei* RUT-C30 mCherry with varying inoculation ratios. Cultivation was conducted in a 48-well round-well microtiter plate within the µRAMOS-BioLector-combination [33, 34]. **a** Calculation of end co-culture ratios according to Eqs. (1) and (2). Spectroscopic measurement settings: integration time = 900 ms, Area 4: 405/580 nm, mCherry: 587/610 nm. The data presented are mean values derived from biological triplicates for each measurement. Error bars indicate the standard deviations. **b** Time point of sampling at the end of the cultivation after 42 h. Images were taken at × 100 magnification. *S. coeruleorubidus* mostly appears in pellets, and *T. reesei* shows dispersed growth

The comparison of the influence of the inoculation rate on the co-culture ratio at the end of the exponential growth phase is shown in Fig. 5a. The axenic culture of *S. coeruleorubidus* (1:0) consists, as expected, 100% of *S. coeruleorubidus*. Similarly, the axenic *T. reesei* culture only contains *T. reesei*, as it was inoculated only with *T. reesei*. The three co-cultures, where *S. coeruleorubidus* was provided in excess (1:0.1, 1:0.2, and 1:0.5), are composed mostly, approximately 80 to 90%, of *S. coeruleorubidus* due to the higher inoculation of this microorganism at the beginning of cultivation. The co-culture with equal OD_start_ for both microorganisms at the beginning of cultivation (1:1) results in 61% *S. coeruleorubidus* and 26% *T. reesei*, meaning that it is still dominated by *S. coeruleorubidus*. This dominance can be explained by the slightly higher maximum growth rate of *S. coeruleorubidus* (0.20 h^−1^ for *S. coeruleorubidus* and 0.16 h^−1^ for *T. reesei*, calculated from logarithmic plot of total oxygen consumption). This effect can also be observed in cultures with a higher OD_start_ of *T. reesei* compared to *S. coeruleorubidus* (0.5:1 and 0.2:1). It is noteworthy that the co-culture with an inoculation ratio of 0.2:1 did not reach a total signal of 100% at the end, instead only achieving 61%. A potential issue in this culture is that the mCherry signal exhibited a distinct, uneven decrease, which could be attributed to fluorescence quenching metabolites. This is supported by the microscopic images, discussed in Fig. 5b, which showed a darker background compared to all other cultivations. Starting at an inoculation ratio of 0.1:1, which significantly favors *T. reesei*, a dominance of this microorganism over *S. coeruleorubidus* is observed, with a ratio of 27 to 56%. To qualitatively verify the results, the findings are compared with microscopic images taken at the end of the cultivation, see Fig. 5b. A look at the two axenic reference cultures reveals that the morphology of *S. coeruleorubidus* is primarily pelleted. The pellets are very dense but do not show clear edges, appearing diffuse and hyphal at the edge. The morphology of *T. reesei*, on the other hand, is more dispersed, with hyphae that, due to their density, appear clumped in the images. Early sporulation of this microorganism is also visible. The microscopic images of the co-cultures showed similar morphology to the axenic cultures with pellets of *S. coeruleorubidus* and dispersed *T. reesei*, suggesting that the two microorganisms do not seem to influence each other’s macromorphology in a co-culture. Co-cultures that, according to the fluorescence measurements, are dominated by *S. coeruleorubidus* (1:0.1, 1:0.2, 1:0.5, 1:1, 1:0.5, and 1:0.2) show a high number of large pellets of *S. coeruleorubidus* as well as dispersed hyphae attributed to *T. reesei*. With increasing *T. reesei* ratio, an increase in dispersed *T. reesei* hyphae can be seen in the images. In the culture dominated by *T. reesei* according to the online signals (0.1:1), the dispersed morphology of *T. reesei* is mainly visible, with just a single, small pellet of *S. coeruleorubidus*. To test the method’s applicability to other *Streptomyces* spp., co-cultures with *S. bobili* were analyzed in the same manner, see Supplement 10 and Supplement 11. Co-cultures inoculated with a higher *S. bobili* ratio exhibited a dominance of this microorganism, whereas co-cultures inoculated with a higher *T. reesei* inoculation ratio were dominated by this species. A nearly balanced co-culture was achieved with an initial inoculation ratio of 1:1, and overgrowth was not observed, consistent with previous results from the *S. coeruleorubidus* co-culture. A qualitative validation of the findings with microscopic images, see Supplement 11b, approved the results of the fluorescence measurements, indicating that the method can be considered reliable and applicable to co-cultures of various *Streptomyces* spp. with *T. reesei*.

Overall, the results demonstrate that the method reliably enables differentiated online monitoring of *Streptomyces* spp. and *T. reesei* in co-culture using the two identified fluorescence wavelengths. Through tracking of growth, varying dynamics were observed across the different cultures. It must of course be noted that this process primarily involves the growth of biomass and no other products are produced in significant amounts, so that the correlation of total oxygen consumption as a measure for biomass is true. For processes with substantial product formation that leads to oxygen consumption, the correlation would no longer be valid. As mentioned in the beginning, the differentiated online monitoring of co-cultures is rarely found in the literature. While established offline methods, such as sequencing or quantitative polymerase chain reaction [21, 22], provide microorganism-specific data, they are limited to discrete time points and cannot capture dynamic, time-resolved courses like our method does. Most reported online methods, such as scattered light measurement, do not allow, or only under certain conditions, for microorganism-specific quantification [22, 24]. Although fluorescence-based measurements have been described in the context of co-culture monitoring, these approaches typically require both microorganisms to be genetically tagged with different fluorescent proteins, as exemplified by the study of Finger et al., who monitored a co-culture by tagging both microorganisms with fluorescent proteins [19]. An interesting approach was employed by Tamura et al., who used two distinct cell lines—a cancerous mutant one and one without cancerous mutations—tagged with fluorescence proteins to monitor the effects of anti-cancer drugs. However, fluorescence was not continuously measured but only after regular sampling followed by fluorescence microscopy [52]. A similar study was conducted by Xin and Yang, who also monitored two different cell lines by fluorescence measurement of two fluorescent protein tags in screening experiments [53]. The results further indicate that varying inoculation ratios lead to different compositions of the co-cultures over time. Cultures with higher OD_start_ of *Streptomyces* spp. lead to considerably higher ratios of *S. coeruleorubidus*, while cultures with higher OD_start_ of *T. reesei* are dominated by *T. reesei*. Since *T. reesei* appears to have a slightly lower maximum growth rate under the set cultivation conditions, it requires a higher inoculation ratio to become the dominant species. However, no complete overgrowth of either microorganism was observed for the tested inoculation ratios, suggesting that none of the tested inoculation ratios provided an extreme advantage for either microorganism and more different inoculation ratios would be required to observe complete overgrowth. This effect, in which the inoculation rate influences the co-culture composition, has already been observed for other co-cultures in the literature. As mentioned in the beginning, Finger et al. investigated co-cultures of one *Streptomyces* sp., specifically *S. coelicolor*, and *T. reesei* on cellulose with varying inoculation ratios. They also observed varying co-culture dynamics depending on the inoculation ratio. No complete overgrowth of one microorganism was observed in their co-culture experiments, even though the inoculation ratio was increased to 1:0.001 [19]. Mellefont et al. also conducted co-culture experiments in which they varied the inoculation ratios of *Escherichia coli*, *Pseudomonas fluorescens*, and *Lactobacillus plantarum* in co-culture with *Listeria monocytogenes*. They observed that with variations in the inoculation ratios, different microorganisms dominated, with the species in higher proportion prevailing. Complete overgrowth was not observed, despite inoculation densities being up to a thousand times higher [54]. In this study, inoculation ratio variation was tested to influence the composition of co-cultures. However, it is also possible to control the composition of co-cultures through other approaches. For example, Finger et al. investigated the variation of osmolality and power input, as one of the microorganisms responded more strongly to the induced stress [19]. Another approach, tested by Wu et al., is time-staggered inoculation. In their study, *S. coelicolor* and *Aspergillus niger* were used in co-culture, where the slower-growing strain was inoculated first, and the other microorganism was introduced later to achieve simultaneous growth [55].

In summary, a novel approach is demonstrated that enables differentiated co-culture online monitoring for simplified application in future experiments. By using different inoculation ratios, the approach was thoroughly tested and proven to be reliable and consistent within the conducted measurements. Additionally, the approach was validated through qualitative data from microscopic images. For future co-culture development, this method can facilitate the screening of co-cultures, providing valuable insights into their dynamics.

## Conclusion

A major challenge in co-culture research is the differentiated monitoring of the different microorganisms. In this study, a fluorescence-based method was successfully developed that enables differentiated co-culture online monitoring, using co-cultures of *Streptomyces* spp. and *T. reesei*. In this approach, *T. reesei* was tagged with the fluorescence protein mCherry, while *Streptomyces* spp. were used as untagged strains. First, 2D fluorescence spectra were recorded, which showed significant differences between the two microorganisms of the co-culture. This allows the identification of promising fluorescence areas for differentiated online monitoring in co-cultures. Time-resolved online monitoring of distinct wavelength combinations in the identified areas revealed correlations between fluorescence intensity and biomass growth. These wavelength combinations, particularly the autofluorescence at 405/480 nm for *Streptomyces* spp. and the mCherry fluorescence of the protein tag at 587/610 nm for *T. reesei*, were then applied for online monitoring of co-cultures. In co-culture, reliable and differentiated online monitoring of those microorganisms was achieved, which was qualitatively confirmed by microscopy images recorded at the end of the cultivation. Additionally, the method was applied to different inoculation ratios, leading to varying co-culture dynamics and compositions, showing its utility for screening experiments of co-cultures. The method was successfully applied to *S. coeruleorubidus* and *S. bobili*. Since the differential 2D fluorescence spectra were similar for other *Streptomyces* spp., this suggests that the method could be extended to other co-cultures involving different *Streptomyces* spp., as well as to entirely different co-culture systems. However, this remains to be validated through further experiments. To enhance the sustainability of the process, it could be further developed to use complex substrates such as cellulose, which *T. reesei* is capable of metabolizing. However, additional research is needed to evaluate the online monitoring method’s applicability under these conditions. Overall, the developed method for differentiated online monitoring shows great potential for future use in co-culture research, including applications like high-throughput screening.

## Supplementary Information

Below is the link to the electronic supplementary material.

Supplementary file 1 (DOCX 3.57 MB)

## Data Availability

Additional data supporting the findings of this study are available in Supplementary Information.

## Abbreviations

µRAMOS-BioLector-combination: In-house-built device that combines a BioLector system and measurement of the respiration activity
2D: Two-dimensional
FAD: Flavin adenine dinucleotide
HPLC: High‐performance liquid chromatography
NADH: Nicotinamide adenine dinucleotide
OD_start_: Optical density at the beginning of the cultivation
OTR: Oxygen transfer rate
*S. bobili*: *Streptomyces bobili*
*S. coelicolor*: *Streptomyces coelicolor*
*S. coeruleorubidus*: *Streptomyces coeruleorubidus*
*Streptomyces * sp./spp.: *Streptomyces* species
*T. reesei*: *Trichoderma reesei*
