# Supplementary material for: Fluorescence-Based Online Monitoring Enables Differentiation in Co-Cultures of Untagged Streptomyces Species and Trichoderma reesei
Source: Appl Biochem Biotechnol. 2025 Sep 9;197(11):7276–95. doi: 10.1007/s12010-025-05378-y (PMC12634738; doi:10.1007/s12010-025-05378-y)
Supplement: Supplementary file 1 — (DOCX 3.57 MB) [file 12010_2025_5378_MOESM1_ESM.docx]

Supplementary Information


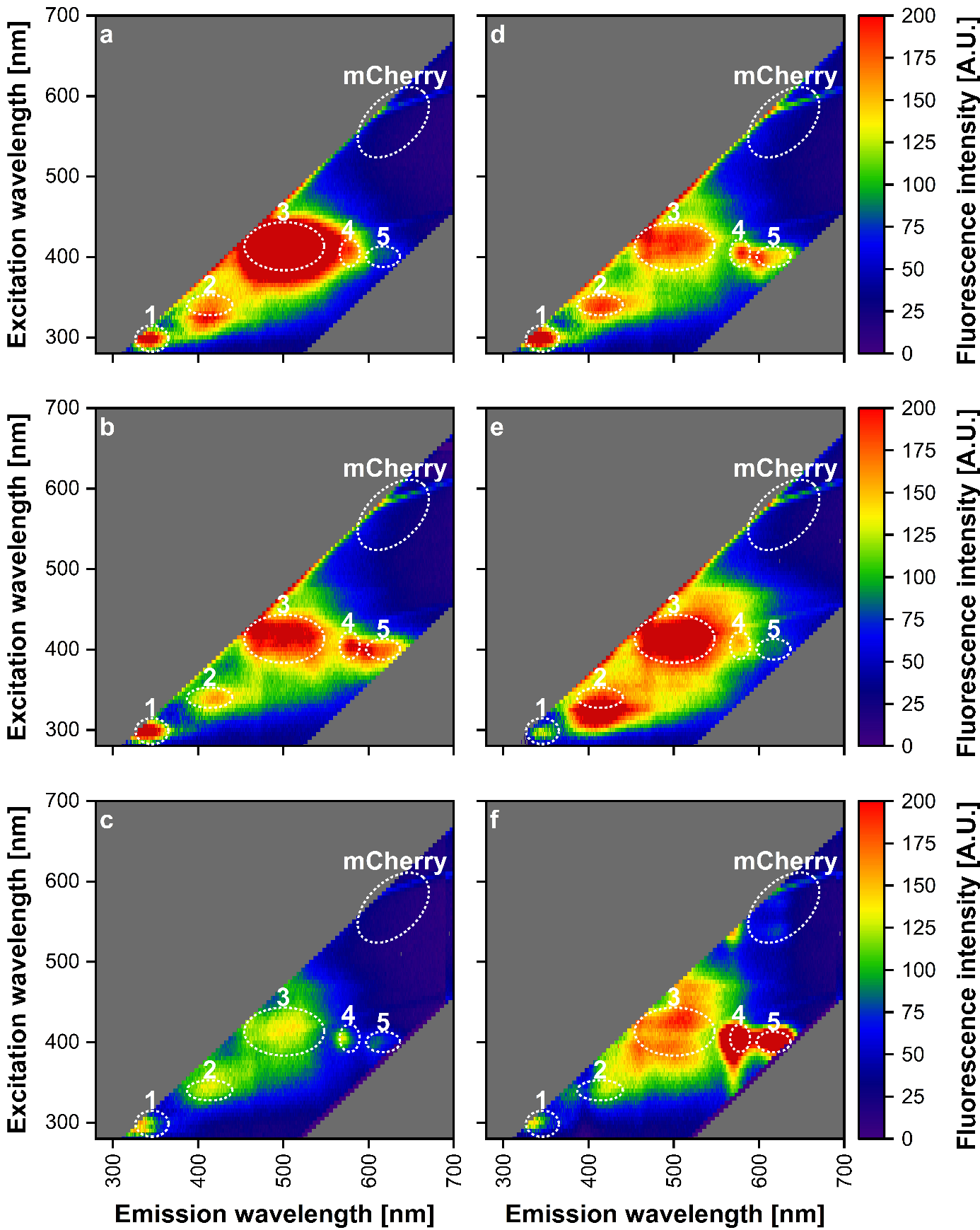
Supplement 1: Differential 2D fluorescence spectra of *Streptomyces albofaciens* (a), *Streptomyces alboniger* (b), *S. bobili* (c), *Streptomyces fradiae* (d), *Streptomyces rimosus* (e), and *Streptomyces venezuelae* (f).

Display of fluorescence intensity depending on different excitation and emission wavelength combinations as contour plot. The spectra were recorded at the end of the growth phase shortly before glucose of the respective cultivation was depleted. Cultivation was conducted in a 48-well round-well microtiter plate within the in-house-built device that measures fluorescence intensity of all excitation and emission wavelength combinations in a defined range [32]. Spectroscopic measurement settings: excitation wavelength range = 270 – 700 nm, excitation step size = 5 nm, emission wavelength range = 270 – 700 nm, emission step size = 1 nm, integration time = 900 ms. The area 30 nm around the scattered light (same excitation and emission wavelength) was cut off. Data was referenced to the first measuring cycle at the beginning of the cultivation. Unmeasured areas are shown in gray. Selected Areas 1 to 5 and mCherry are marked with white, dotted circles.


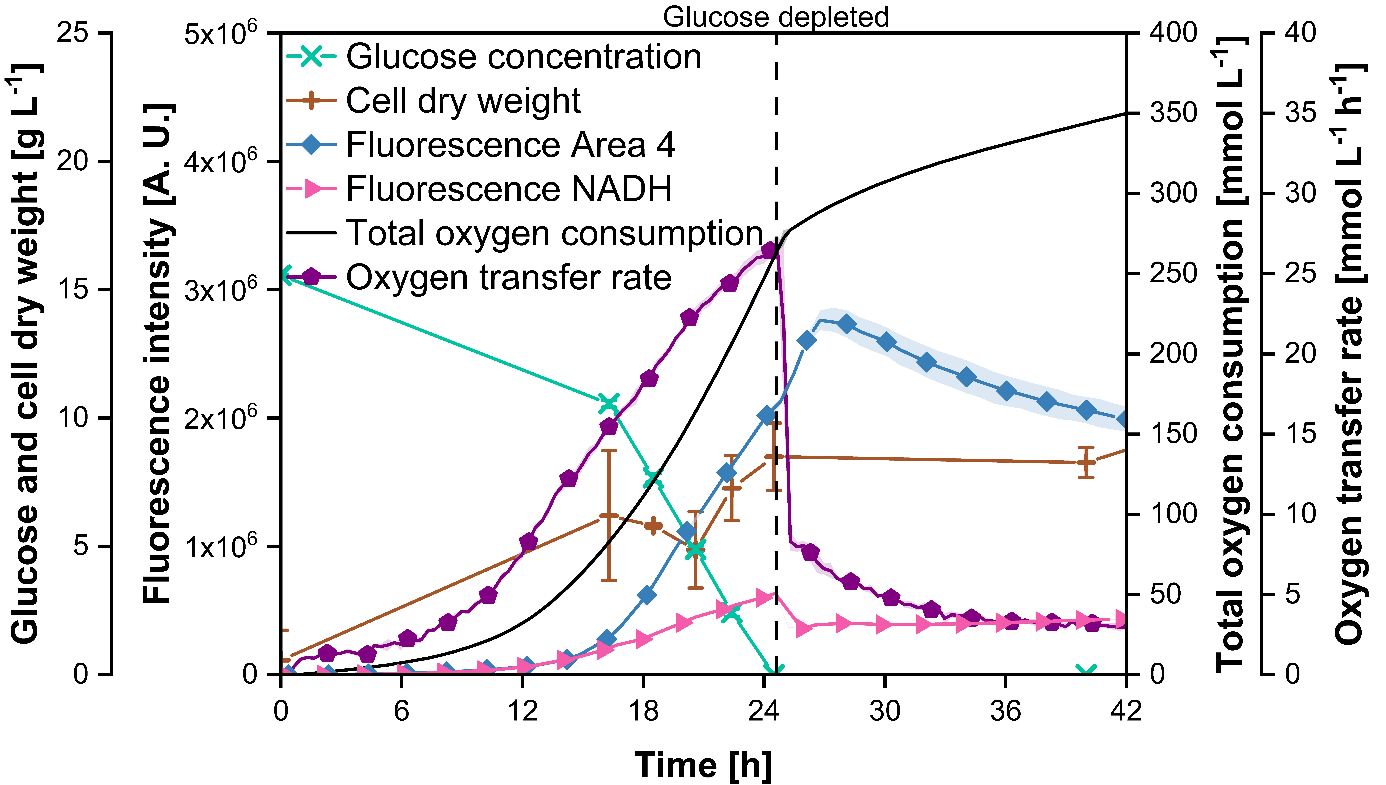
Supplement 2: Determining the time point of glucose depletion by comparing different online and offline measurement methods.

Cultivation was conducted in a 48-well round-well microtiter plate within the µRAMOS-BioLector-combination [33, 34] with *S. coeruleorubidus*. Spectroscopic measurement settings: integration time = 900 ms, Area 4: 405/580 nm, NADH: 340/460 nm. For clarity, only every third data point of fluorescence intensity and every sixth data point for OTR is plotted as a symbol, respectively. Lines are drawn through all data points. The shaded areas indicate the standard deviations. Glucose concentration was measured using HPLC, and cell dry weight was determined gravimetrically. Every measured data point is displayed, and error bars indicate the standard deviations. The data presented are mean values derived from biological triplicates for each measurement. The time point of the end of growth due to glucose depletion is displayed by a vertical dashed line.


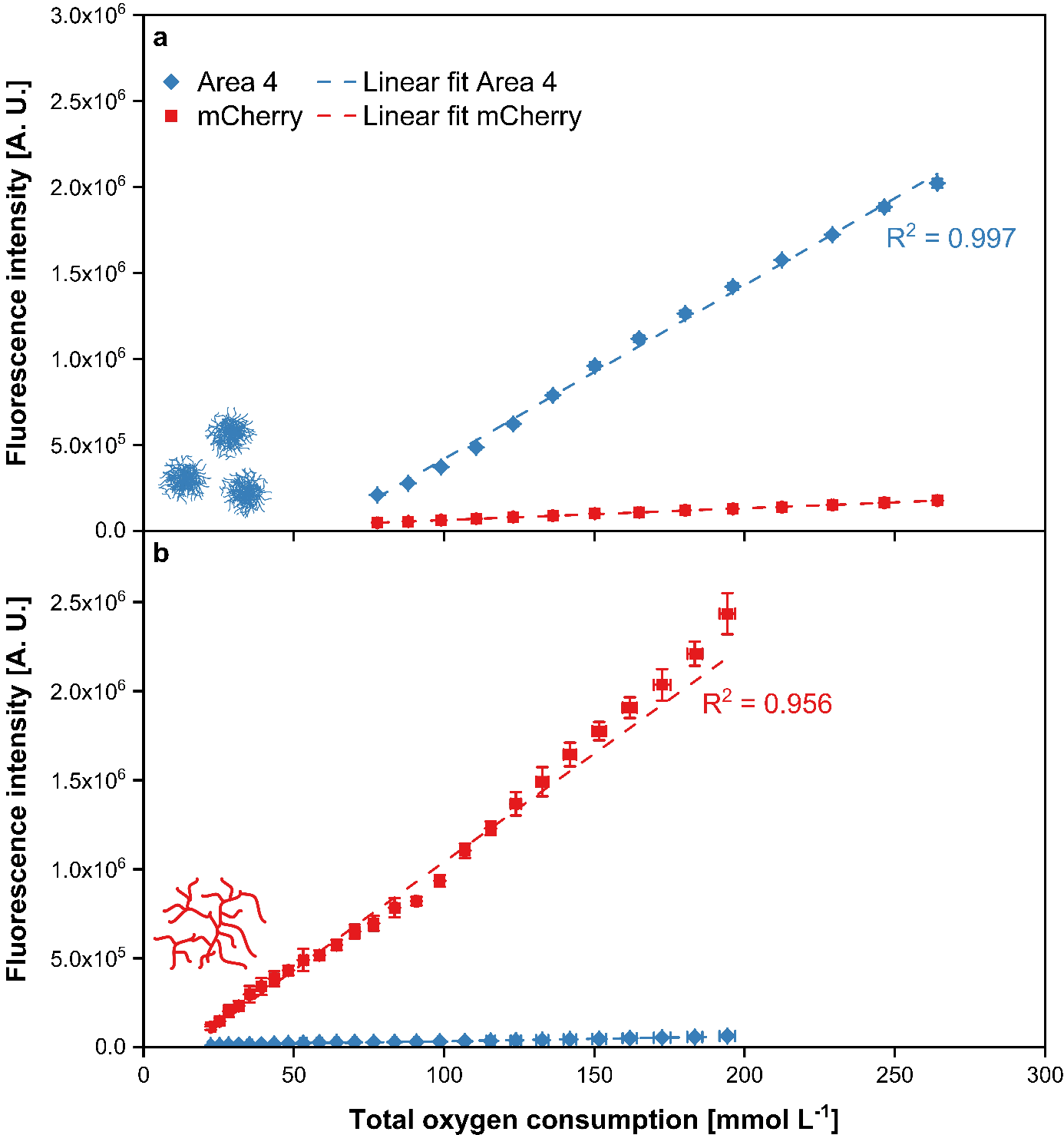
Supplement 3: Correlation of fluorescence intensity of selected fluorescence wavelength combinations against total oxygen consumption of axenic cultures of *S. coeruleorubidus* (a) and *T. reesei* RUT‑C30 mCherry (b).

Cultivation was conducted in a 48-well round-well microtiter plate within the µRAMOS-BioLector-combination [33, 34]. Spectroscopic measurement settings: integration time = 900 ms, Area 4: 405/580 nm, mCherry: 587/610 nm. The data presented are mean values derived from biological triplicates for each measurement. Error bars in both the x- and y-axis directions indicate the standard deviations. Only data of the growth phase until glucose depletion is shown and was used for the regression.


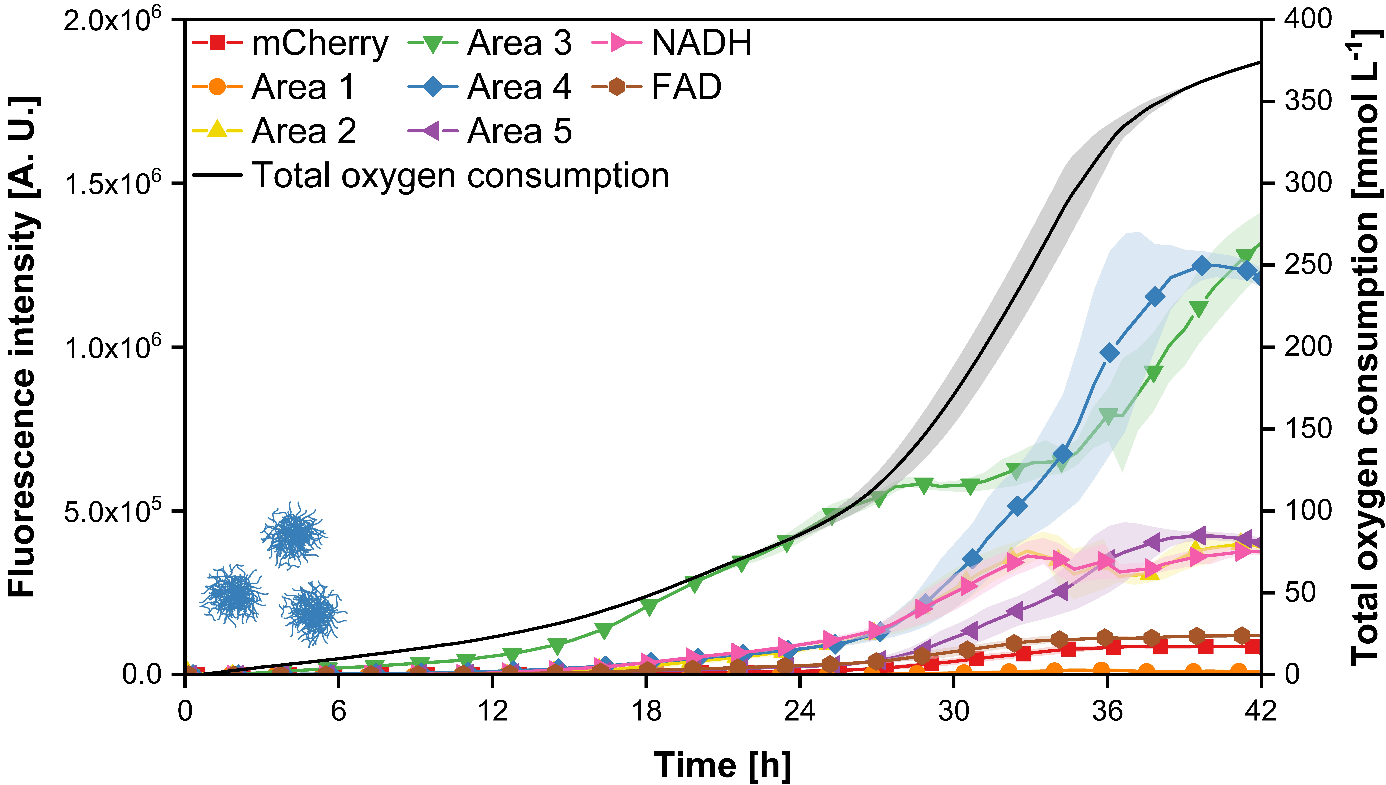
Supplement 4: Time-resolved fluorescence measurement of selected fluorescence wavelength combinations of axenic cultures of *S. bobili*.

Cultivation was conducted in a 48-well round-well microtiter plate within the µRAMOS-BioLector-combination [33, 34]. Spectroscopic measurement settings: integration time = 900 ms, Area 1: 280/350 nm, Area 2: 340/420 nm, Area 3: 400/470 nm, Area 4: 405/580 nm, Area 5: 400/630 nm, NADH: 340/460 nm, FAD: 340/530 nm, mCherry: 587/610 nm. For clarity, only every third data point of fluorescence intensity is plotted as a symbol. Lines are drawn through all data points. The data presented are mean values derived from biological triplicates for each measurement (except for the total oxygen consumption data, which are derived from a duplicate). The shaded areas indicate the standard deviations (or the minimum and maximum values for the duplicate).


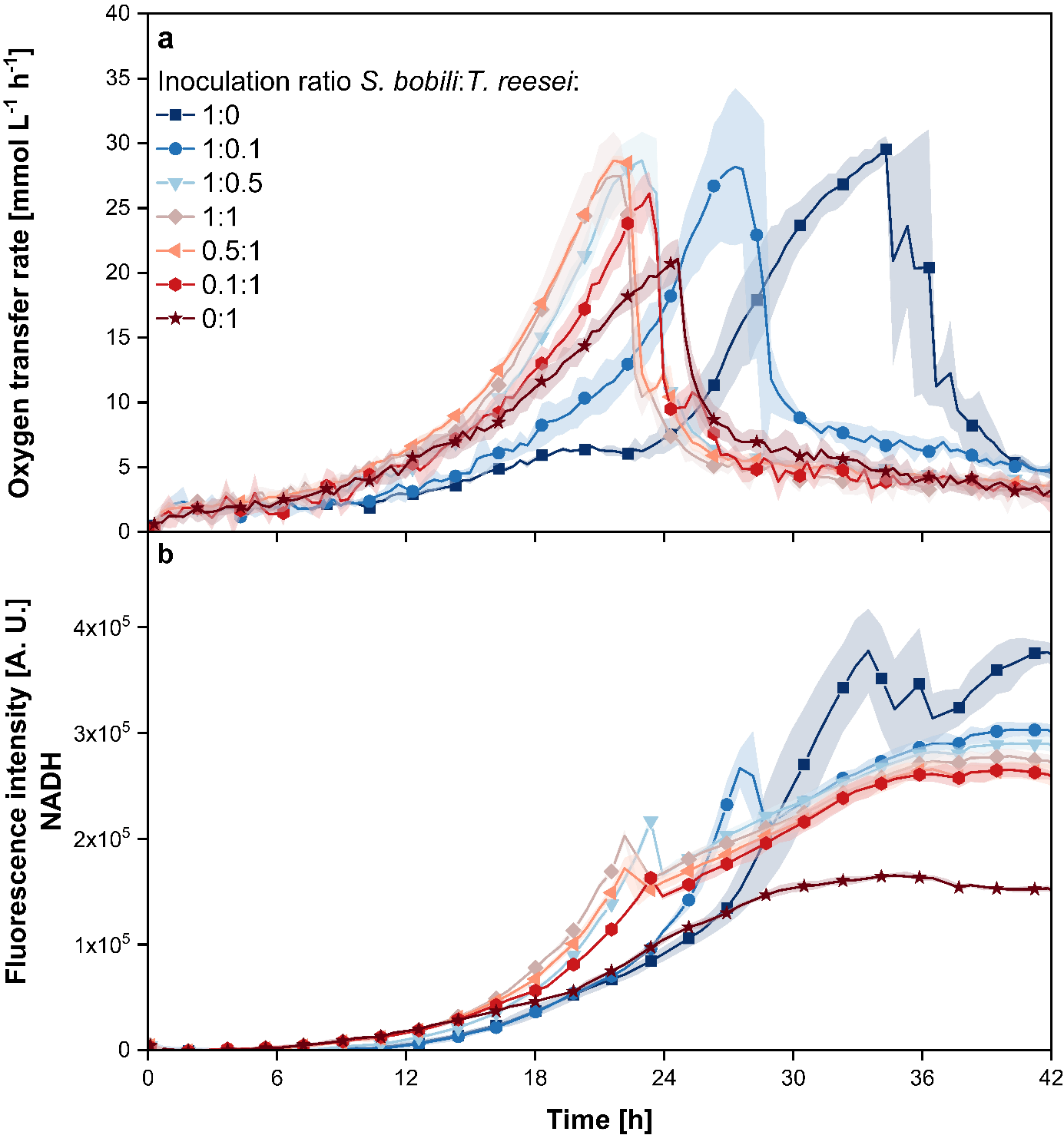
Supplement 5: Time-resolved OTR (a) and fluorescence measurement of NADH (b) of co-cultures of *S. bobili* and *T. reesei* RUT‑C30 mCherry with varying inoculation ratios.

Cultivation was conducted in a 48-well round-well microtiter plate within the µRAMOS-BioLector-combination [33, 34]. Spectroscopic measurement settings: integration time = 900 ms, NADH: 340/460 nm. For clarity, only every third data point of fluorescence intensity and every sixth data point for OTR is plotted as a symbol. Lines are drawn through all data points. The data presented are mean values derived from biological triplicates for each measurement (except for the OTR data of the inoculation ratio 1:0, which are derived from a duplicate). The shaded areas indicate the standard deviations (or the minimum and maximum values for the duplicate).


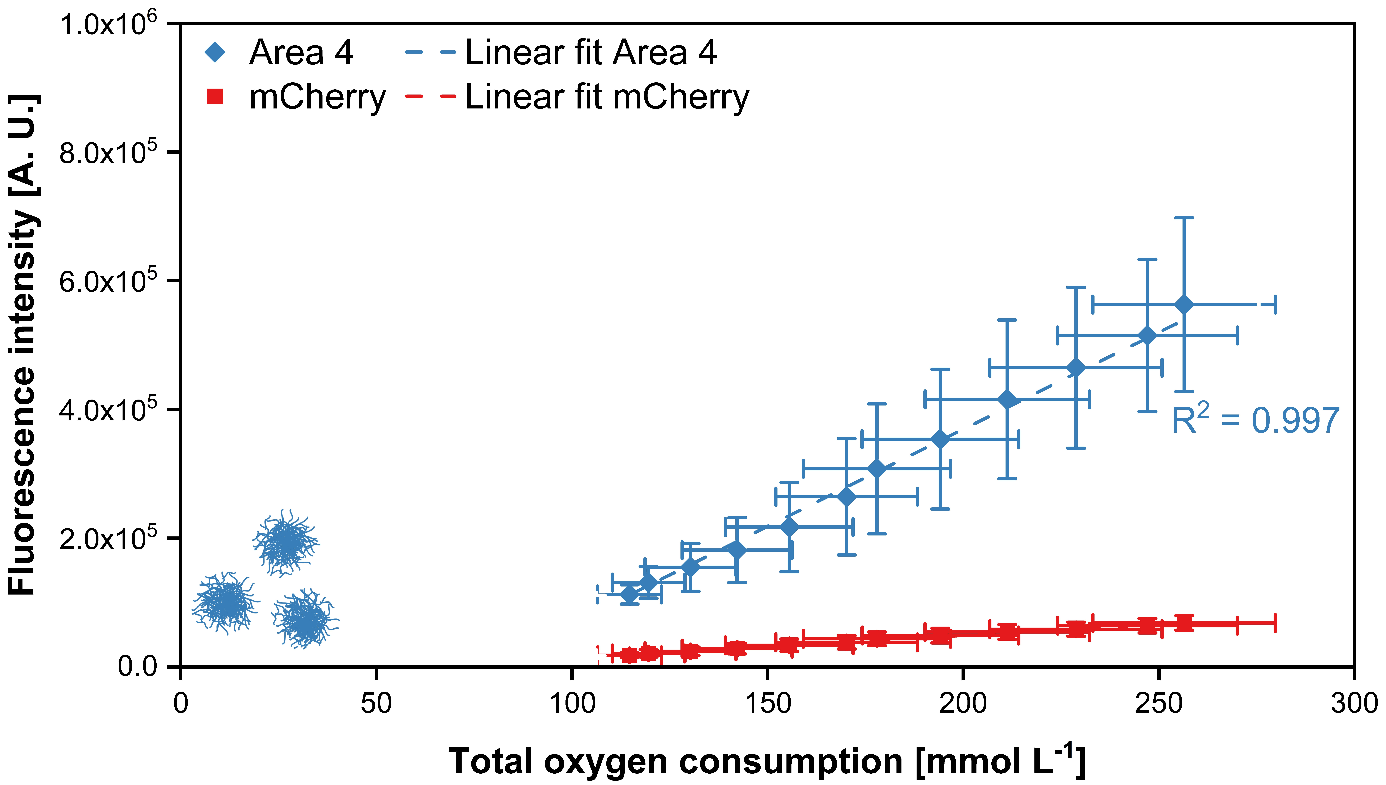
Supplement 6: Correlation of fluorescence intensity of selected fluorescence wavelength combinations against total oxygen consumption of axenic cultures of *S. bobili*.

Cultivation was conducted in a 48-well round-well microtiter plate within the µRAMOS-BioLector-combination [33, 34]. Spectroscopic measurement settings: integration time = 900 ms, Area 4: 405/580 nm, mCherry: 587/610 nm. The data presented are mean values derived from biological triplicates for each measurement (except for the total oxygen consumption data, which are derived from a duplicate). Error bars in both the x- and y-axis directions indicate the standard deviations (or the minimum and maximum values for the duplicate). Only data of the growth phase until glucose depletion is shown and was used for the regression.


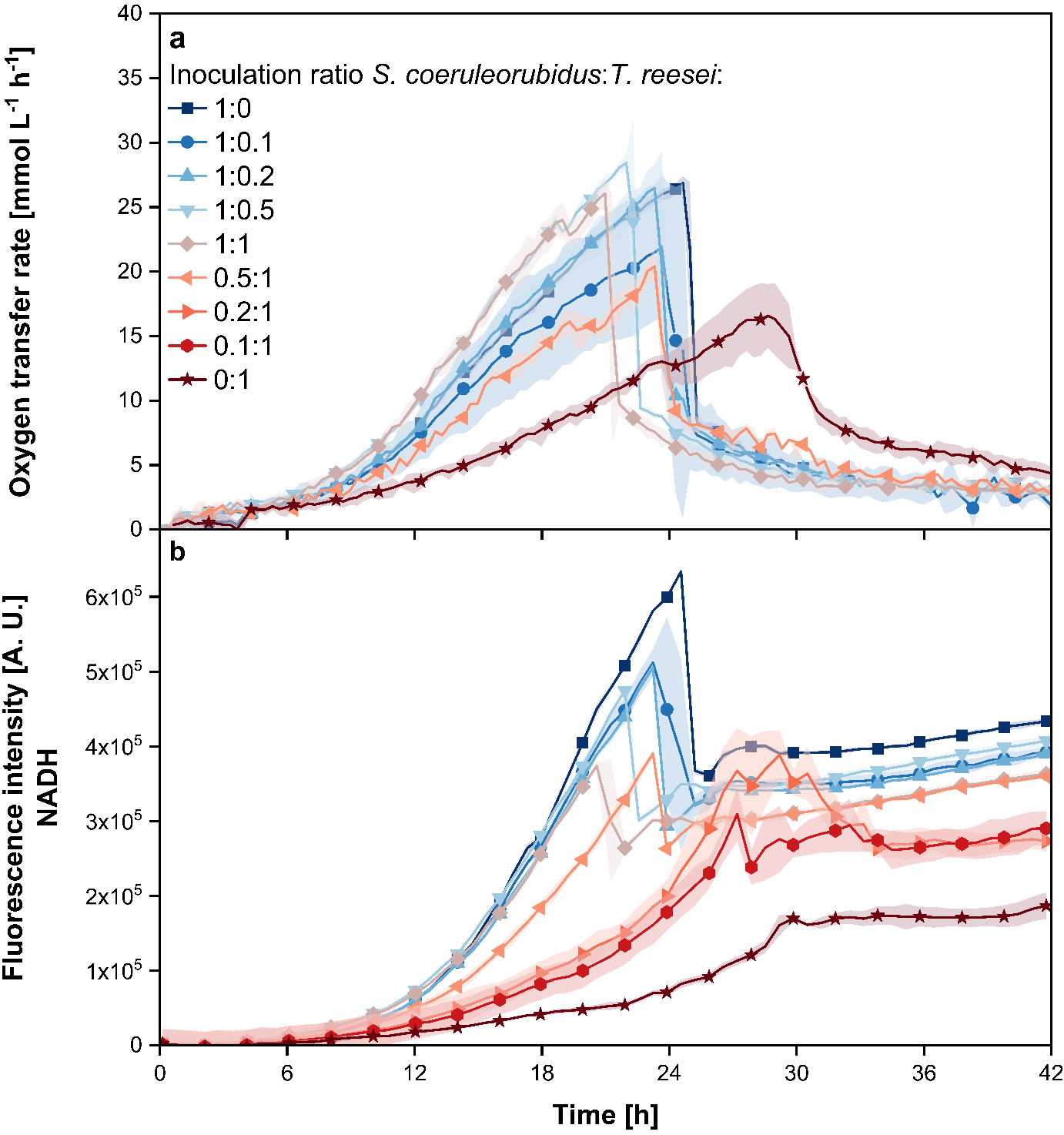
Supplement 7: Time-resolved OTR (a) and fluorescence measurement of NADH (b) of co-cultures of *S. coeruleorubidus* and *T. reesei* RUT‑C30 mCherry with varying inoculation ratios.

Cultivation was conducted in a 48-well round-well microtiter plate within the µRAMOS-BioLector-combination [33, 34]. Spectroscopic measurement settings: integration time = 900 ms, NADH: 340/460 nm. For clarity, only every third data point of fluorescence intensity and every sixth data point for OTR is plotted as a symbol. Lines are drawn through all data points. The data presented are mean values derived from biological triplicates for each measurement (except for the OTR data of the inoculation ratio 0.5:1, which are derived from a duplicate). The shaded areas indicate the standard deviations (or the minimum and maximum values for the duplicate). OTR data of 0.2:1 and 0.1:1 are missing due to measurement error.


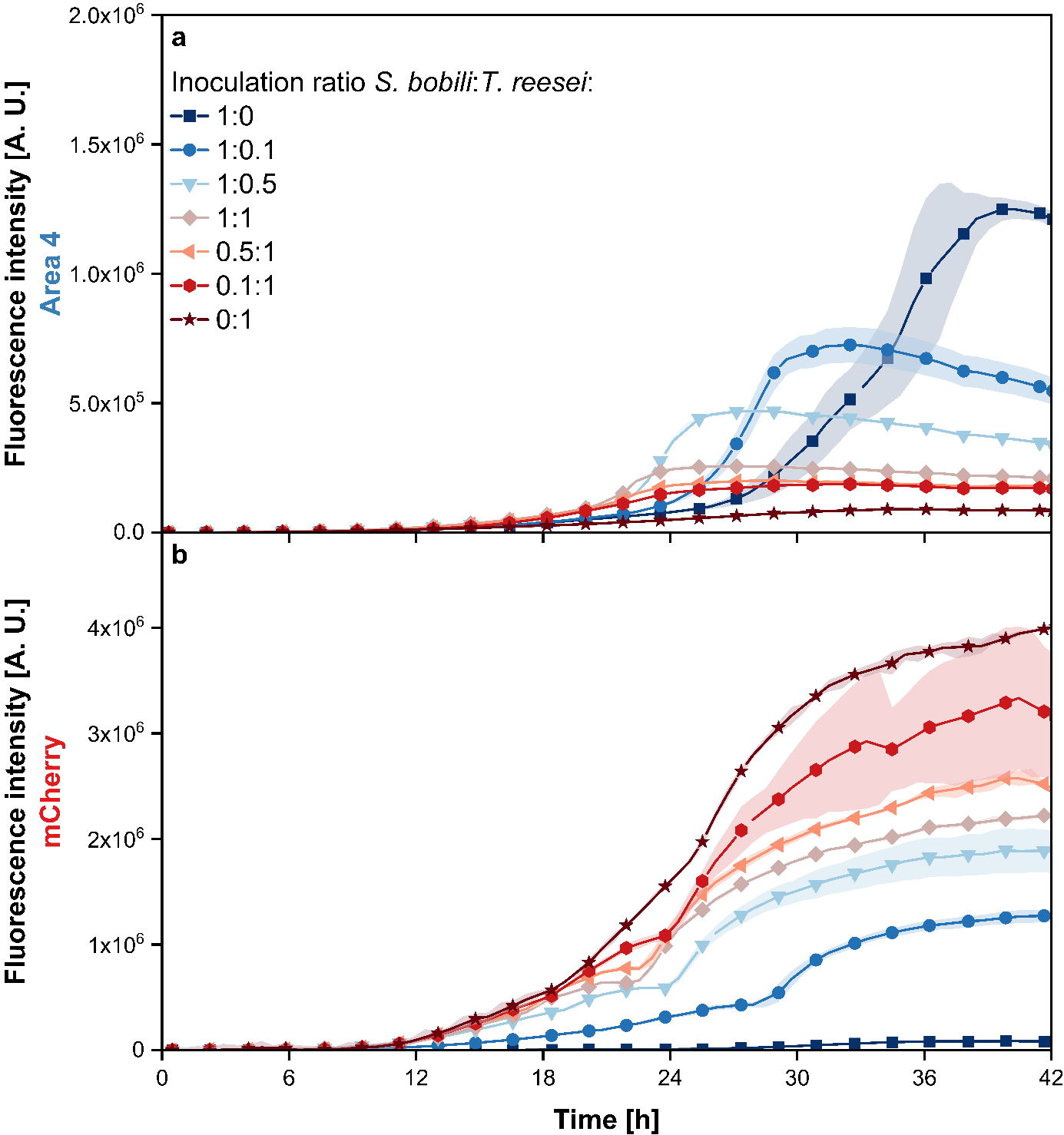
Supplement 8: Time-resolved fluorescence measurement of Area 4 (a) and mCherry (b) of co-cultures of *S. bobili* and *T. reesei* RUT‑C30 mCherry with varying inoculation ratios.

Cultivation was conducted in a 48-well round-well microtiter plate within the µRAMOS-BioLector-combination [33, 34]. Spectroscopic measurement settings: integration time = 900 ms, Area 4: 405/580 nm, mCherry: 587/610 nm. For clarity, only every third data point is plotted as a symbol. Lines are drawn through all data points. The data presented are mean values derived from biological triplicates for each measurement. The shaded areas indicate the standard deviations.


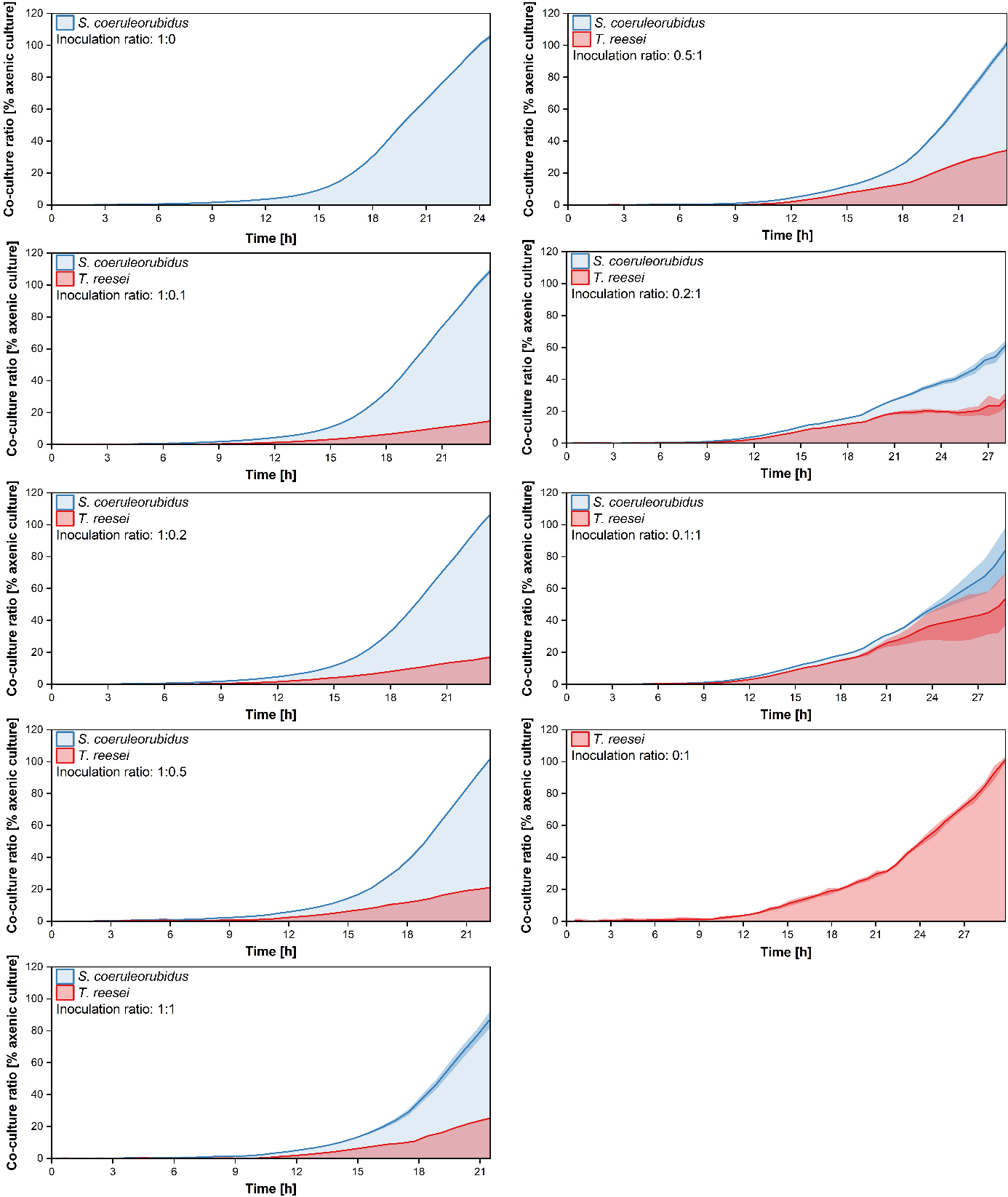
Supplement 9: Time-resolved co-culture ratio compared to axenic cultures for co-cultures of *S. coeruleorubidus* and *T. reesei* RUT‑C30 mCherry with varying inoculation ratios.

Calculation of co-culture ratios according to Equations (1) and (2). Cultivation was conducted in a 48-well round-well microtiter plate within the µRAMOS-BioLector-combination [33, 34]. Spectroscopic measurement settings: integration time = 900 ms, Area 4: 405/580 nm, mCherry: 587/610 nm. The data presented are mean values derived from biological triplicates for each measurement. The shaded areas indicate the standard deviations. Only data of the growth phase until glucose depletion is shown.


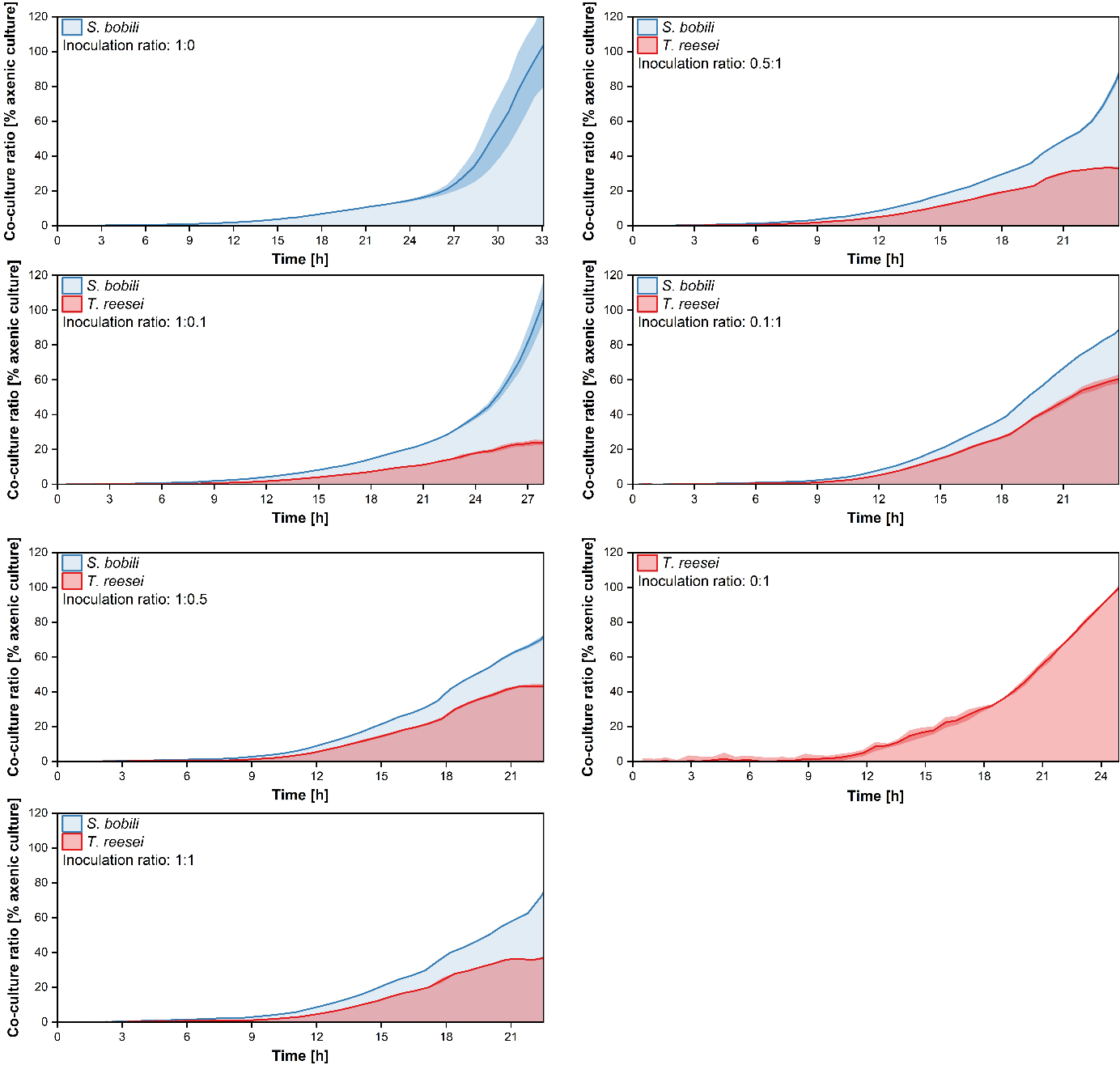
Supplement 10: Time-resolved co-culture ratio compared to axenic cultures for co-cultures of *S. bobili* and *T. reesei* RUT‑C30 mCherry with varying inoculation ratios.

Calculation of co-culture ratios according to Equations (1) and (2). Cultivation was conducted in a 48-well round-well microtiter plate within the µRAMOS-BioLector-combination [33, 34]. Spectroscopic measurement settings: integration time = 900 ms, Area 4: 405/580 nm, mCherry: 587/610 nm. The data presented are mean values derived from biological triplicates for each measurement. The shaded areas indicate the standard deviations. Only data of the growth phase until glucose depletion is shown.


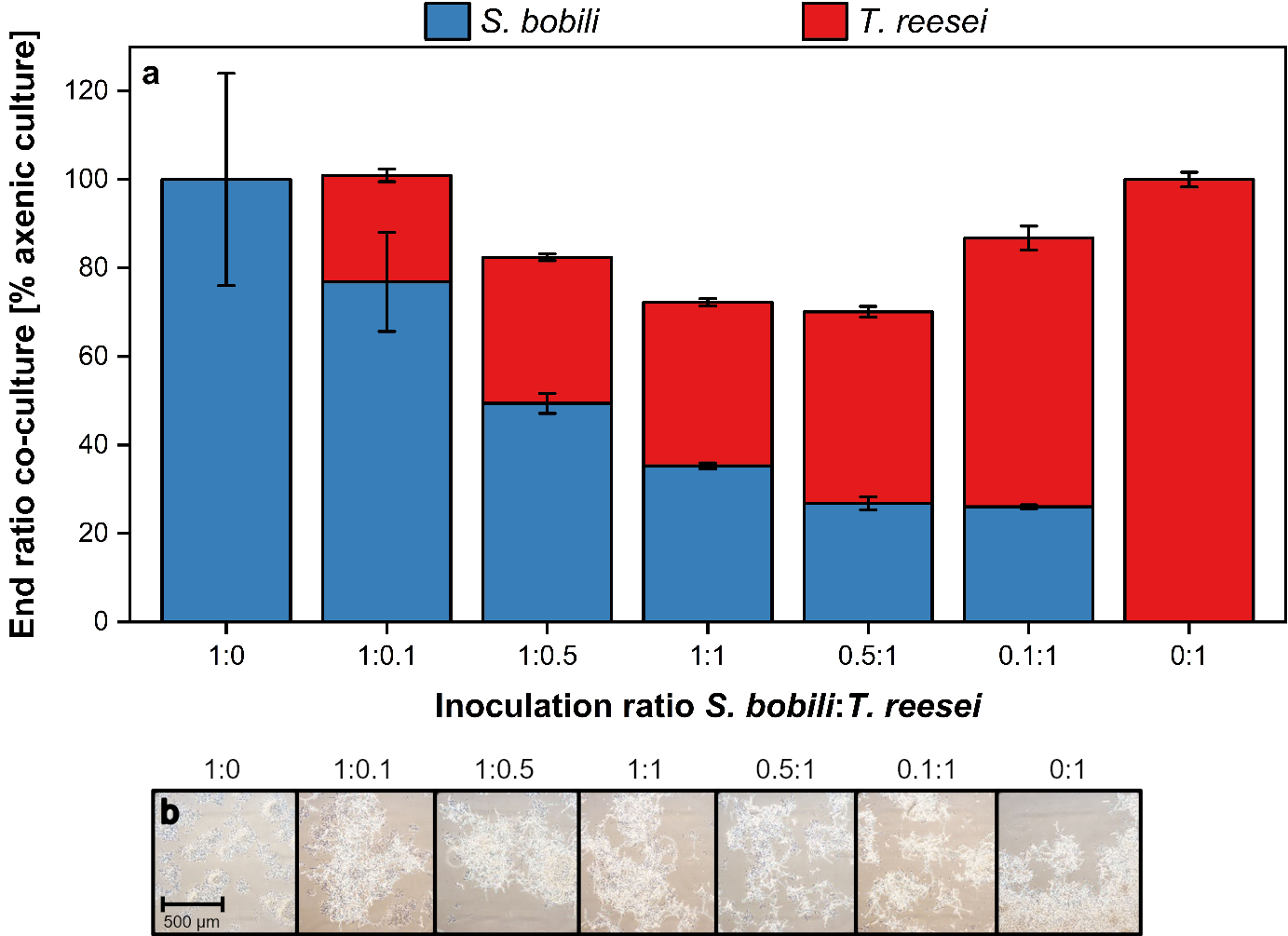
Supplement 11: End ratios (a) and microscopic images (b) of co-cultures of *S. bobili* and *T. reesei* RUT‑C30 mCherry with varying inoculation ratios.

Cultivation was conducted in a 48-well round-well microtiter plate within the µRAMOS-BioLector-combination [33, 34]. a: Calculation of end co-culture ratios according to Equations (1) and (2). Spectroscopic measurement settings: integration time = 900 ms, Area 4: 405/580 nm, mCherry: 587/610 nm. The data presented are mean values derived from biological triplicates for each measurement. Error bars indicate the standard deviations. b: Time point of sampling at the end of the cultivation after 42 h. Images were taken at 100x magnification. *S. bobili* mostly appears in pellets, and *T. reesei* shows dispersed growth.
